# Identifying Targetable Vulnerabilities to Circumvent or Overcome Venetoclax Resistance in Diffuse Large B-Cell Lymphoma

**DOI:** 10.3390/cancers16112130

**Published:** 2024-06-03

**Authors:** Clare M. Adams, Amanda McBride, Peter Michener, Irina Shkundina, Ramkrishna Mitra, Hyun Hwan An, Pierluigi Porcu, Christine M. Eischen

**Affiliations:** 1 Department of Pharmacology, Physiology, and Cancer Biology, Thomas Jefferson University, 233 South 10th St., Philadelphia, PA 19107, USA; 2 Department of Medical Oncology, Sidney Kimmel Cancer Center, Thomas Jefferson University, 834 Chestnut St., Philadelphia, PA 19107, USA

**Keywords:** venetoclax/ABT-199 resistance, B-cell lymphoma, BCL2, mitochondrial electron transport chain (ETC), IDH2

## Abstract

**Simple Summary:**

Diffuse large B-cell lymphoma (DLBCL) is an aggressive blood cancer and the most common non-Hodgkin lymphoma. DLBCL has a 40% relapse rate with current therapies, warranting investigations into potential vulnerabilities leading to new treatment avenues. Many DLBCLs increase the levels of a protein called BCL2 that blocks lymphoma cell death and contributes to treatment resistance and relapse. A drug was developed to target BCL2 (venetoclax/ABT-199) to induce DLBCL cell death. However, it was ineffective as a single agent, revealing that DLBCL relies on more than BCL2 for survival. Our study of intrinsic venetoclax resistance and developed venetoclax resistance in DLBCL identifies new targetable vulnerabilities in DLBCL by exposing dependencies on critical cellular processes. Our findings have the potential to lead to new treatments for DLBCL and possibly other B-cell lymphomas.

**Abstract:**

Clinical trials with single-agent venetoclax/ABT-199 (anti-apoptotic BCL2 inhibitor) revealed that diffuse large B-cell lymphoma (DLBCL) is not solely dependent on BCL2 for survival. Gaining insight into pathways/proteins that increase venetoclax sensitivity or unique vulnerabilities in venetoclax-resistant DLBCL would provide new potential treatment avenues. Therefore, we generated acquired venetoclax-resistant DLBCL cells and evaluated these together with intrinsically venetoclax-resistant and -sensitive DLBCL lines. We identified resistance mechanisms, including alterations in BCL2 family members that differed between intrinsic and acquired venetoclax resistance and increased dependencies on specific pathways. Although combination treatments with BCL2 family member inhibitors may overcome venetoclax resistance, RNA-sequencing and drug/compound screens revealed that venetoclax-resistant DLBCL cells, including those with TP53 mutation, had a preferential dependency on oxidative phosphorylation. Mitochondrial electron transport chain complex I inhibition induced venetoclax-resistant, but not venetoclax-sensitive, DLBCL cell death. Inhibition of IDH2 (mitochondrial redox regulator) synergistically overcame venetoclax resistance. Additionally, both acquired and intrinsic venetoclax-resistant DLBCL cells were similarly sensitive to inhibitors of transcription, B-cell receptor signaling, and class I histone deacetylases. These approaches were also effective in DLBCL, follicular, and marginal zone lymphoma patient samples. Our results reveal there are multiple ways to circumvent or overcome the diverse venetoclax resistance mechanisms in DLBCL and other B-cell lymphomas and identify critical targetable pathways for future clinical investigations.

## 1. Introduction

Diffuse large B-cell lymphoma (DLBCL) is the most common lymphoma subtype in adults. While approximately 60% of DLBCL patients are cured with frontline chemotherapy [1], treatment outcomes in patients with relapsed or refractory disease are very poor [1], highlighting the need for new therapeutic options. DLBCL is a heterogeneous group of B-cell lymphomas with diverse molecular alterations and clinical outcomes. For example, DLBCLs with TP53 tumor suppressor mutations and/or MYC amplification/overexpression are particularly aggressive, chemo-resistant, and associated with lower survival rates [1,2,3]. Two main DLBCL subtypes, germinal center B-cell-like (GCB) and activated B-cell (ABC), are clinically recognized [4,5], but recent analyses have identified further molecular subtypes, underscoring the complexity of DLBCL [6,7,8,9]. However, one common theme in DLBCL is the ability to evade apoptosis through dysregulation of the mitochondrial apoptosis pathway. This pathway is governed by the BCL2 protein family through tightly regulated interactions between the five anti-apoptotic (BCL2, BCLX, BCLW, MCL1, and BFL1) and the many pro-apoptotic family members [10]. Alterations that dysregulate this pathway lead to increased survival, promote lymphomagenesis, and contribute to treatment resistance [10,11].

BCL2 is frequently overexpressed in DLBCL through translocation (GCB subtype), constitutive NF-ĸB activation (ABC subtype), or less frequently by BCL2 amplification [12,13,14,15]. High BCL2 expression is an independent predictor of poorer outcomes in DLBCL [16,17]. Moreover, dysregulated BCL2 is a hallmark of more aggressive subsets of DLBCL, such as those that have both BCL2 and MYC rearrangements (originally called double-hit DLBCL), now classified as DLBCL high-grade B-cell lymphoma (HGBL), which are typically GCB subtype [18]. There are also aggressive DLBCLs that have elevated expression of both BCL2 and MYC not due to rearrangement that are called double expressors and are typically ABC DLBCL [1,19]. Thus, BCL2 was a logical therapeutic target in DLBCL. However, single-agent therapy with the BCL2-specific inhibitor venetoclax (ABT-199, PubChem 254741640) had only a 12% complete response (CR) rate in relapsed/refractory DLBCL and 14% CR in follicular lymphoma in a phase I study [20]. While efficacious combinations with venetoclax have been identified and FDA-approved for other hematologic malignancies, including AML [21,22] and CLL with 1p deletion [23], early-phase studies have not yet identified optimal therapeutic combinations in DLBCL [24,25]. To that end, uncovering DLBCL mechanisms of resistance to venetoclax will provide critical insight into its therapeutic application in DLBCL.

To date, several mechanisms of venetoclax resistance have been elucidated in AML, CLL, and mantle cell lymphoma, but studies in DLBCL are limited and do not explain inherent resistance [26,27,28]. Here, we demonstrate that venetoclax resistance mechanisms in DLBCL are diverse. Through analysis of DLBCL cells with acquired or intrinsic venetoclax resistance, we identified several ways to overcome or circumvent venetoclax resistance in DLBCL. Combination treatment with venetoclax and MCL1 inhibition could overcome both acquired and intrinsic venetoclax resistance. Inhibition of CDK7/9, B-cell receptor signaling, or class I HDACs effectively killed venetoclax-resistant DLBCL cells. Importantly, we determined that venetoclax-resistant DLBCLs, including those with TP53 mutation, were sensitive to mitochondrial electron transport chain (ETC) inhibition. Notably, we identified a new approach to overcome venetoclax resistance by combining inhibition of isocitrate dehydrogenase 2 (IDH2) with venetoclax, which was synergistic. Our data highlight multiple potential and new therapeutic avenues for venetoclax-resistant DLBCL treatment that may also be efficacious in other B-cell malignancies. 

## 2. Materials and Methods

### 2.1. DLBCL Cell Lines

SUDHL2, SUDHL4, SUDHL5, SUDHL6, SUDHL8, SUDHL10, SUDHL16, and Toledo cell lines were purchased from and cultured as described by ATCC. OCI-Ly3 and OCI-Ly10 cell lines were obtained from Dr. John Chan while at the University of Nebraska Medical Center. OCI-Ly3 cells were cultured in RPMI (Gibco, Thermo Fisher Scientific Inc., Waltham, MA, USA) with 20% FBS, 1% L-glutamine, and 1% penicillin/streptomycin. OCI-Ly10 cells were cultured in IMDM (Gibco), 20% human AB serum (Valley Biomedical, Winchester, VA, USA), 0.1 mg/mL ascorbic acid, 15 μg/mL D-ribose, and 1% penicillin/streptomycin. Cell lines were authenticated by short tandem repeat profiling and were routinely tested for mycoplasma using MycoSensor PCR Assay Kit (Agilent, Santa Clara, CA, USA). The specific DLBCL subtypes of the cell lines used are listed in Supplementary Table S1.

### 2.2. Generation of Venetoclax-Resistant Cell Lines

Venetoclax-resistant DLBCL cell lines were generated by chronic exposure to gradually increasing concentrations of venetoclax over 7–13 months, starting at sub-lethal venetoclax concentrations (~IC10), which was the concentration of venetoclax at which 10% of the cells were killed by 48 h. Cell lines were considered venetoclax-resistant when they maintained viability and grew in culture with continuous exposure to venetoclax at a concentration of at least 10× the original IC50, which was the concentration of venetoclax at which 50% of the cells were killed by 48 h. Venetoclax-resistant lines were maintained in venetoclax to keep venetoclax pressure. For experiments, parental and venetoclax-resistant DLBCL cells were resuspended in fresh media.

### 2.3. Inhibitors and Drug/Compound Screen

Protein inhibitors were purchased from vendors, and AbbVie (North Chicago, IL, USA) provided a portion of the BCL2 family inhibitors (Supplementary Table S1). For the drug/compound screen, venetoclax-resistant and its matched venetoclax-sensitive parental line were screened at The Wistar Institute Molecular Screening and Protein Expression Facility (https://www.wistar.org/resources/molecular-screening-protein-expression-facility/). Lines were screened for their sensitivity to 1919–2240 drugs/compounds (see Supplementary Materials for details). Estimated IC50 values were determined using a nonlinear regression fit of the data to a one-site dose–response equation using XLFit5 (IDBS, Woking, UK). For analysis, the geometric means of the IC50 values were calculated for compounds that were duplicated. Compounds with small structural modifications or different formulations with similar IC50 values were grouped, and if IC50 values were different, they were not grouped. Fold-changes in IC50 values of each compound/grouped compounds were calculated by comparing resistant to parental lines. Those that showed approximately 1.5-fold or greater sensitivity were divided according to the pathways they target (see Supplementary Materials).

### 2.4. Cell Survival and Synergy Analyses

Cells were placed in 96-well round bottom plates (10,000–50,000 cells/well depending on the line’s rate of growth, triplicates/quadruplicates). Following 48 h incubation with targeted inhibitors, cell survival/viability was measured using CellTiter-96 Aqueous One Solution Cell Proliferation Assay (MTS assay, 492 nm; Promega, Madison, WI, USA) or CellTiter-Fluor Cell Viability Assay (Promega). Single-agent IC50 concentrations were determined by nonlinear regression of log (inhibitor) vs. normalized response analysis and plotted using the curve-fitting guides for dose–response curves in GraphPad Prism (version 10.0.0). For determination of synergy, cells were treated with at least 3–4 concentrations of each inhibitor, both as a single agent and in combination, with doses ranging between the approximate IC10 and IC50. Synergy scores were calculated using SynergyFinderPlus online tool [29]. Synergy was defined as a synergy score > 10, according to the ZIP method, but additional methods (Bliss, HSA, Loewe) were also used for further verification (see Supplementary Materials). 

### 2.5. Western Blotting

Whole-cell protein lysates were generated using RIPA buffer (50 mM Tris pH 7.4, 150 mM NaCl, 1% sodium deoxycholate, 1% Triton X-100, 0.1% SDS), and equal amounts of protein from each sample were compared by Western blot and chemiluminescence was detected by film. The antibodies used are in Supplementary Table S1.

### 2.6. Caspase-3/7 Apoptosis Analysis

DLBCL cells were incubated with inhibitors in triplicate, and percentages of apoptotic cells were determined by detection of activated Caspase-3/7 using either the CellEvent Caspase-3/7 Green Flow Cytometry Assay Kit (Invitrogen, Waltham, MA, USA) for DLBCL cell lines and one marginal zone lymphoma patient sample or the CellEvent Caspase-3/7 Green Detection Reagent for plate-based assays (Invitrogen) for the rest of the patient samples, according to the manufacturer’s instructions. Flow cytometry was performed on LSRII or Celesta cytometers and analyzed using FlowJo software (version 10.8.0, BD Biosciences, Franklin Lakes, NJ, USA).

### 2.7. Live/Dead Assay

Cells were placed in 96-well round bottom plates (10,000–50,000 cells/well depending on the line’s rate of growth, triplicates), and live/dead assay (Abcam, Cambridge, UK) was performed according to the manufacturer’s instructions for flow cytometric analysis at 72 or 96 h after adding compounds. Samples were run on a Fortessa cytometer (BD Biosciences) and analyzed with FlowJo software.

### 2.8. RNA-Sequencing and Data Availability

Total RNA was isolated using TRIzol (Ambion, Thermo Fisher Scientific Inc.) following the manufacturer’s protocol. RNA concentration and integrity/quality were determined by Qubit (Thermo Fisher Scientific Inc.) and Agilent TapeStation, respectively. RNA-sequencing was performed by Azenta Life Sciences (Burlington, MA, USA) and Thomas Jefferson University Genomics core facility. RNA-sequencing profiles for SUDHL6 and SUDHL16 parental and venetoclax-resistant cell lines were generated using Illumina HiSeq 4000 and NovaSeq 6000 platforms, respectively. Libraries were prepared using the NEBNext Ultra II RNA Library Preparation Kit for SUDHL6 (New England Biolabs, Ipswich, MA, USA) and the Illumina Stranded Total RNA Prep with RiboZero Plus ligation for SUDHL16 (Illumina, San Diego, CA, USA). Evaluation of RNA-sequencing data and follow-up functional analyses were conducted using standard pipelines (see Supplementary Materials). Data are available in GEO (GSE252306). 

### 2.9. Sequencing BCL2, BAX, and TP53

Total RNA was isolated as described above. cDNA was generated using the SuperScript III First-strand Synthesis System (Invitrogen) prior to PCR amplification and sequencing using the following primers: BCL2-Forward 5′-CGTCCAAGAATGCAAAGCAC and BCL2-Reverse 5′-TCATGGTACATCACTGACAATGC; BAX-Forward 5′-AGCGGCGGTGATGGACG; and BAX-Reverse 5′-ACCCCTCCCAGAAAAATGCC. We previously published the *TP53* primers [30]. PCR amplicons were separated by gel electrophoresis, extracted, and submitted for Sanger sequencing at Azenta Life Sciences.

### 2.10. Flow Cytometry Intracellular Protein Analysis

Single-cell suspensions were incubated with fluorophore-linked antibodies against specific proteins (extracellular or intracellular) or isotype controls (Supplementary Table S1). Fluorophore-positive cells were determined based on isotype controls. For cell lines, only intracellular staining was performed. For patient lymphoma samples, viability staining followed by surface staining was performed prior to intracellular staining. For viability, the fixable viability stain 440UV (BD Biosciences) was used per the manufacturer’s protocol. For surface staining, cells were re-suspended in FACS buffer (PBS + 0.5% BSA) containing a cocktail of surface antibodies. For antibody cocktails containing 2 or more antibodies conjugated to BV fluorescent dyes, Brilliant Stain Buffer (BD Biosciences) was used instead of FACS buffer. For intracellular staining of anti-apoptotic BCL2 proteins, cells were first fixed with 4% paraformaldehyde, then permeabilized with 0.1% Triton X-100 in PBS, and finally re-suspended in FACS buffer containing a cocktail of antibodies specific for BCL2 family proteins. Samples were evaluated on a Fortessa cytometer (BD Biosciences) and analyzed using FlowJo software. Patient samples were gated on the B-cell markers CD20+, CD19+, Kappa+, and/or Lambda+ for analysis.

### 2.11. Patient Samples

Fresh (not FFPE), de-identified tissue was obtained, following written informed consent, from surgical biopsies of patients with suspected aggressive lymphoma through the CAP-certified Sidney Kimmel Cancer Center Biorepository at Thomas Jefferson University under IRB#20D.826. All samples showed viability of >90%. Data are shown from samples with a confirmed diagnosis of DLBCL, follicular lymphoma, or marginal zone lymphoma following hematopathological review. Patient characteristics and other details (e.g., further diagnosis classification, cytogenetic features, etc.) are listed in Supplementary Table S2. Tissue samples were processed into single-cell suspensions by gentle mechanical disruption through a 100 μm cell strainer. Intracellular protein analysis was performed as described above. For survival assays, B-cells were isolated using the Human B-cell Isolation Kit II (Miltenyi Biotec, Bergisch Gladbach, Germany). The number and viability of recovered cells were determined using Trypan Blue Dye exclusion. For cell survival analyses using ETC and IDH2 inhibitors, cell survival was determined following 24 h of treatment using the CellTiter-Fluor Cell Viability Assay (Promega). All other cell viability assays were completed using CellTiter-96 Aqueous One Solution Cell Proliferation Assay (Promega), as described above. Caspase-3/7 activity was measured as described above.

### 2.12. Statistics

Experiments were performed with at least three technical triplicates for each sample and at least two independent experiments were performed per condition for each cell line. Data are presented as mean ± standard deviation (SD) or standard error of the mean (SEM) and are indicated in the figure legends. Statistical significance was determined using unpaired, two-tailed Student *t*-tests when comparing two groups or one-way ANOVA with Bonferroni multiple comparisons test when comparing more than two groups at a single time point using GraphPad Prism software (version 10.0.0). Significance for RNA-seq data with read counts was determined using edgeR (version 3.38.4) as described in the Supplementary Materials, and Hallmark pathway enrichment analysis was based on an FDR < 0.05 cutoff. Statistical significance of differential gene expression was based on a *p*-value cutoff of 0.05 after being adjusted by the Benjamini–Hochberg multiple test correction method with at least a 1.5-fold change.

## 3. Results

### 3.1. Differences in BCL2 Family Member Expression in Intrinsically Venetoclax-Sensitive and -Resistant DLBCL Cells

**Figure 1:**
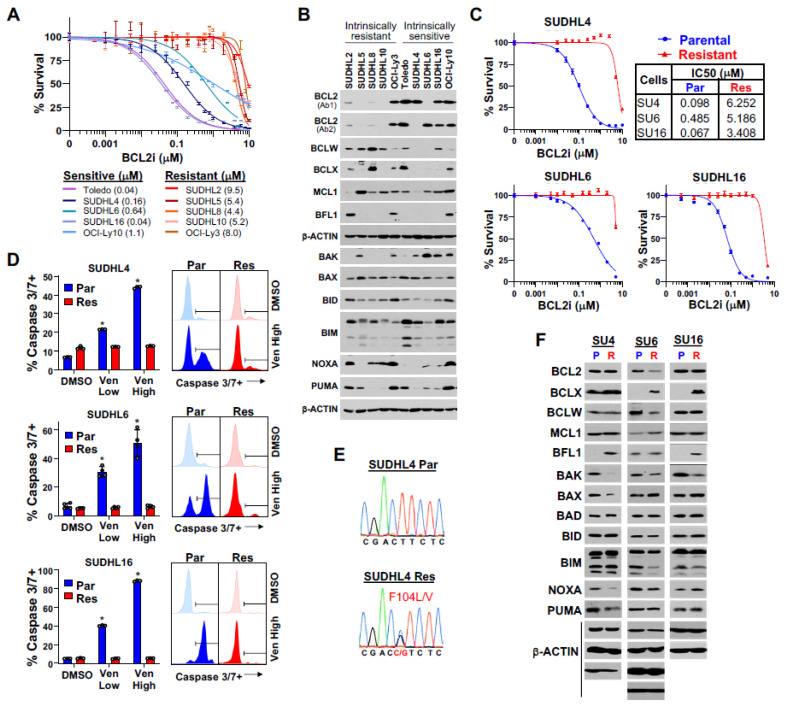
Generation and characterization of DLBCL cell lines with acquired venetoclax resistance. (**A**) Venetoclax dose–response curves (MTS, 48 h, relative to DMSO vehicle control, quadruplicates, mean ± SEM) for DLBCL cell lines with intrinsic venetoclax sensitivity (blue shades) or resistance (red shades). IC50 values in parentheses. (**B**) Western blots of the indicated proteins from the 10 DLBCL lines in A. Each β-actin blot is associated with the blots above it. Two different BCL2 antibodies were needed to detect BCL2 in SUDHL4 and SUDHL6. (**C**) Venetoclax dose–response curves of parental (Par, blue) and acquired venetoclax-resistant (Res, red) DLBCL lines (MTS, 48 h, relative to DMSO vehicle control, quadruplicates, mean ± SEM). IC50 values of each indicated. (**D**) Caspase-3/7 activity after 12 h of venetoclax (Ven) treatment or DMSO vehicle control of the lines from C (triplicates/quadruplicates, mean ± SD). Low and high doses of venetoclax are the IC50 and 10× the IC50 of the parental line, respectively. Representative histograms following high-dose venetoclax treatment or DMSO shown. * *p* < 2.30 × 10^−5^, compared to vehicle (DMSO) control. (**E**) Chromatograms of *BCL2* sequencing of the SUDHL4 parental and acquired venetoclax-resistant lines. (**F**) Western blots of BCL2 family members of the three parental (P) and acquired venetoclax-resistant (R) DLBCL lines (BCL2 Ab1 used for SU4 and SU16; BCL2 Ab2 used for SU6); note that some exposures in (**F**) were longer than in (**B**) to detect lower-expressed proteins. Each β-actin blot is associated with at least one of the blots above it.

To gain insight into the contribution of BCL2 to DLBCL survival, we screened ten DLBCL lines that included GCB and ABC subtypes for sensitivity to venetoclax. We identified two groups, one with sensitivity to venetoclax (IC50 0.04–1.1 µM) and the other with intrinsic resistance (IC50 4.4–9.5 µM) to venetoclax (Figure 1A). Moreover, BCL2 protein levels in these lines showed that venetoclax sensitivity tended to correlate with higher BCL2 protein expression (Figure 1B), as previously reported [31,32]. However, OCI-Ly3 cells demonstrated intrinsic venetoclax resistance despite high BCL2 levels, indicating that factors beyond BCL2 expression contribute to venetoclax sensitivity. Due to amino acid differences at antibody binding sites in two venetoclax-sensitive DLBCL lines, SUDHL4 and SUDHL6, two BCL2 antibodies were needed to detect BCL2 (Figure 1B, Supplementary Table S1). 

Comparing BCL2 family member expression showed that DLBCL lines with intrinsic venetoclax resistance had higher levels of BCLW, whereas venetoclax-sensitive lines had higher levels of BCL2 (Figure 1B), suggesting that a reciprocal relationship may exist between the two. Venetoclax-sensitive lines also typically had increased pro-apoptotic BAK levels (Figure 1B), which is a key effector of mitochondrial pore formation for apoptosis [33]. BCLX was overexpressed to varying degrees in half of all lines, irrespective of venetoclax sensitivity/resistance. MCL1 overexpression was evident in all but two DLBCL lines, but BFL1 was overexpressed in only three lines (ABC subtype) and did not correlate with venetoclax sensitivity/resistance (Figure 1B). Evaluation of the levels of pro-apoptotic BCL2 family members, other than BAK, did not show any clear correlation with venetoclax sensitivity or resistance (Figure 1B). Together, these data suggest that high expression of BCLW alone or together with BCLX or MCL1 and lower levels of BAK may confer venetoclax resistance. Furthermore, BCL2 and BAK overexpression distinguish venetoclax-sensitive DLBCL lines. Therefore, protein levels of specific BCL2 family members were indicative of intrinsic sensitivity or resistance to venetoclax and indicated that BCL2/BAK and BCLW levels are potential biomarkers for sensitivity/resistance.

### 3.2. BCL2 Family Member Alterations in Acquired Venetoclax Resistance in DLBCL Cells

To study mechanisms of acquired venetoclax resistance in DLBCL, we generated three venetoclax-resistant DLBCL lines from SUDHL4, SUDHL6, and SUDHL16 lines with increasing venetoclax concentration exposure over many months. Two of the lines (SUDHL4 and SUDHL6) harbored both *BCL2* and *MYC* translocations [34]. DLBCL lines with acquired venetoclax resistance (≥10× their original IC50; Figure 1C) maintained viability following exposure to a log-fold higher dose of venetoclax, compared to their parental counterparts, which showed venetoclax-dose-dependent apoptosis (Figure 1D). 

To investigate the causes of acquired venetoclax resistance in the DLBCL lines, we took multiple approaches. We first sequenced *BCL2*, as acquired *BCL2* mutations in and around the BH3-binding domain can diminish venetoclax binding, leading to resistance [35,36,37]. There were acquired heterozygous mutations (F104L/V) in the BH3-binding domain of *BCL2* only in SUDHL4 cells (Figure 1E; Supplementary Table S3), suggesting at least a partial dependence on BCL2 for survival. Only SUDHL6 had reduced BCL2 protein levels (Figure 1F). We also sequenced the pro-apoptotic BCL2 family member *BAX* that mediates the apoptotic signal, as *BAX* mutations are reported in CLL and AML patients treated with venetoclax [38,39]. *BAX* was not mutated in any of the acquired venetoclax-resistant DLBCL lines (Supplementary Table S3), but its protein levels were reduced in SUDHL4 cells. In addition, although parental DLBCL lines with *TP53* mutations (e.g., SUDHL4, SUDHL6, and SUDHL16) were not more resistant to venetoclax than those with wild-type *TP53* (e.g., SUDHL5 and OCI-Ly10), *TP53* mutation is associated with reduced venetoclax sensitivity in AML and CLL [40,41]. Of the three venetoclax-sensitive parental DLBCL lines, none gained a new *TP53* mutation following acquired venetoclax resistance (Supplementary Table S3). Therefore, neither *BAX* nor *TP53* mutations contributed to acquired DLBCL venetoclax resistance in the DLBCL lines, but a BH3-binding domain mutation in *BCL2* in SUDHL4 cells did. Also, reduced levels of BCL2 in SUDHL6 cells may have impacted venetoclax sensitivity.

We next assessed if acquired venetoclax resistance was associated with altered levels of BCL2 family members, as we detected in the intrinsically venetoclax-resistant lines and as previously suggested in venetoclax-treated CLL patients and in pre-clinical models of AML and other hematologic malignancies [35,37,42,43]. There were increased BCLX levels in both SUDHL6 and SUDHL16, the two lines that did not have a BCL2 BH3-domain mutation, and SUDHL6 or SUDHL16 also had increased MCL1 or BFL1, respectively (Figure 1F). Additionally, similar to the intrinsically venetoclax-resistant DLBCL lines, there was a decrease in BAK protein in all three acquired venetoclax-resistant DLBCL lines (Figure 1F), which has an essential role in executing apoptosis and is typically not evaluated [10]. Therefore, reduced BAK and increased BCLX were reoccurring changes in acquired venetoclax resistance, although there was heterogeneity in the expression of other BCL2 family members that may also contribute to venetoclax resistance development in DLBCL. Unexpectedly, the BH3-domain mutant SUDHL4 cells also had increased BFL1 and reduced pro-apoptotic BAX, NOXA, and PUMA, which also likely contributed to their venetoclax resistance.

### 3.3. Overcoming Venetoclax Resistance in DLBCL Cells with Combination BCL2 Family Member Inhibition

**Figure 2:**
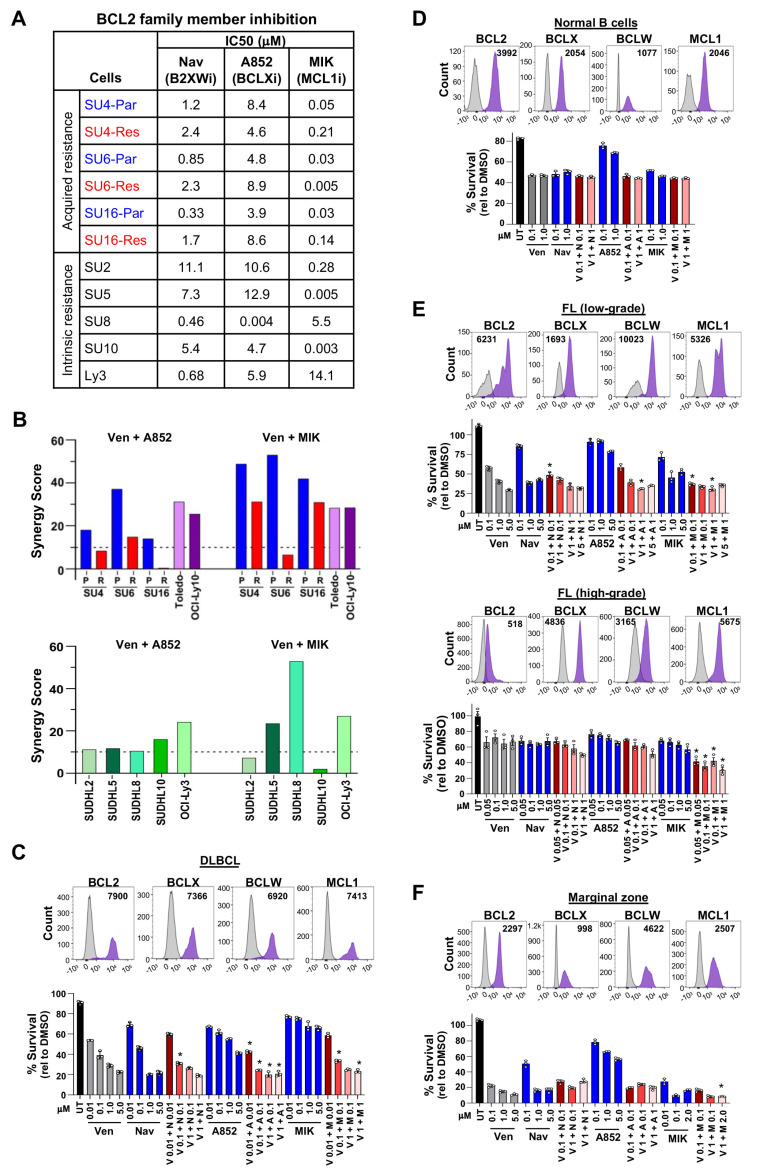
Combination treatment with BCL2 family member inhibitors is effective for DLBCL, follicular, and marginal zone lymphomas. (**A**) IC50s (μM) of acquired venetoclax-resistant (Res) and parental (Par) DLBCL lines and intrinsically venetoclax-resistant DLBCL lines following 48 h of treatment with navitoclax (Nav, B2XWi), A-1331852 (A852, BCLXi), or MIK665 (MIK, MCL1i). (**B**) ZIP synergy scores of venetoclax-sensitive and acquired venetoclax-resistant DLBCL lines (top) or intrinsically venetoclax-resistant (bottom) treated with venetoclax (Ven, BCL2i) + A852 (BCLXi) or MIK (MCL1i). See Supplementary Table S4 for synergy scores from other synergy methods. (**C**–**F**) Intracellular flow cytometry of four anti-apoptotic BCL2 family members and treatment results with venetoclax (Ven, V), navitoclax (Nav, N), A-1331852 (A852, A), MIK665 (MIK, M), or untreated (UT) in fresh patient samples of DLBCL (**C**), normal B-cells (**D**), follicular lymphoma (**E**), and marginal zone lymphoma (**F**). Representative histograms shown with median fluorescence intensity (MFI) after subtracting the isotype control MFI value. Following B-cell enrichment, cell survival (MTS, relative to DMSO vehicle control, triplicates, mean ± SD) was measured 6–12 h after treatment with the compounds indicated. For (**C**) * *p* < 0.01, (**E**) * *p* < 0.05 (top) and * *p* < 0.01 (bottom), and (**F**) * *p* < 0.05, comparing each concentration used in the combination treatment to the same concentration of each single agent.

Given that one or more BCL2 family member levels changed with venetoclax resistance, we evaluated sensitivity to navitoclax (targets BCL2, BCLX, and BCLW) and a BCLX-specific inhibitor (A-1331852) [44], as well as an MCL1-specific inhibitor (MIK665) [45]. All the acquired venetoclax-resistant lines had an expected increased resistance to navitoclax (Figure 2A, Supplementary Figure S1A), which has greater affinity for BCL2 than BCLX and BCLW [46]. Surprisingly, the significantly increased BCLX levels in the venetoclax-resistant SUDHL6 and SUDHL16 lines (Figure 1F) did not correlate with increased sensitivity to BCLX inhibition (Figure 2A, Supplementary Figure S1A); however, it did for BCLX-overexpressing intrinsically venetoclax-resistant SUDHL8 cells (Figure 1B and Figure 2A, Supplementary Figure S1B). The BH3-domain mutant SUDHL4 cells were more sensitive to BCLXi but were significantly more resistant to MCL1i than their parental line (Figure 2A, Supplementary Figure S1A), providing further evidence that additional apoptosis-inhibiting changes than just the BH3-domain mutation in BCL2 have occurred. SUDHL16 venetoclax-resistant cells were also more resistant to MCL1i than their parental line. In contrast, the SUDHL6-resistant line was significantly more sensitive to MCL1i (Figure 2A, Supplementary Figure S1A), and this was consistent with its increased MCL1 levels (Figure 1F). The intrinsically venetoclax-resistant SUDHL5 and SUDHL10 cells that have the highest levels of MCL1 (Figure 1B) were very sensitive to MCL1 inhibition; however, SUDHL2 cells that had low levels of MCL1 were also sensitive to MCL1 inhibition (Figure 2A, Supplementary Figure S1B). Without a specific inhibitor for BCLW or BFL1, it is unclear how reliant on these proteins the venetoclax-resistant lines are. Overall, these data indicate that upregulation of alternate anti-apoptotic BCL2 proteins in intrinsic or acquired venetoclax-resistant cells can, but does not consistently, correlate to increased dependence on those proteins and likely is due to changes in pro-apoptotic BCL2 family members, such as BAK, and proteins in other pathways. Additionally, the observation of heterogeneous alterations in BCL2 family proteins and a lack of consistency between expression and sensitivity to inhibition between resistant lines suggests that multiple mechanisms of venetoclax resistance develop in DLBCL.

Given that we previously reported that DLBCL patient samples [47], as well as cell lines in this current study (Figure 1B,F), typically overexpress more than one anti-apoptotic BCL2 family member, and in light of the low clinical efficacy of single-agent venetoclax in DLBCL [20], we assessed whether the addition of BCLX or MCL1 inhibitors to venetoclax was able to sensitize DLBCL cells to venetoclax. Viability was measured after combination treatment with BCLXi or MCL1i plus venetoclax (BCL2i) at a range of doses (Supplementary Figure S1C–E). Synergy analyses showed a synergistic effect of both drug combinations in all parental lines and the other intrinsically venetoclax-sensitive lines (Figure 2B, Supplementary Figure S1C,D, Supplementary Table S4). However, MCL1i + BCL2i was only synergistic in SUDHL4 (BH3-domain mutant) and SUDHL16 acquired venetoclax-resistant lines (Figure 2B, Supplementary Figure S1C, Supplementary Table S4). Across the five DLBCL lines with intrinsic venetoclax resistance, MCL1i + BCL2i showed a synergistic effect in three lines (Figure 2B, Supplementary Figure S1E, Supplementary Table S4). BCLXi + BCL2i was additive or barely synergistic for the acquired and inherently venetoclax-resistant lines, except OCI-Ly3, for which it was synergistic (Figure 2B, Supplementary Figure S1C,E, Supplementary Table S4). Also, any synergy observed in the acquired venetoclax-resistant cells was less robust (lower synergy scores) compared to parental counterparts (Figure 2B, Supplementary Table S4). Collectively, MCL1 inhibition together with venetoclax was sufficient most of the time to overcome both acquired and intrinsic venetoclax resistance, but BCLX inhibition rarely did.

### 3.4. Combination Treatment of DLBCL, Follicular, and Marginal Zone Lymphoma Patient Samples with BCL2 Family Inhibitors

To gain further understanding of whether BCL2 family member levels correlated with sensitivity to BCL2 family inhibitors alone and in combination, we next evaluated patient samples. Because of the limited sample size, we utilized intracellular protein analysis of anti-apoptotic BCL2 family members by flow cytometry. To determine the accuracy of this method and the antibodies chosen, we tested the levels of intracellular BCL2, BCLX, BCLW, and MCL1 protein in DLBCL cell lines that had high or low levels of the specific protein by Western blot (Figure 1B). Intracellular analysis of BFL1 could not be performed due to the lack of a sufficiently BFL1-specific antibody. The intracellular levels of each protein matched the Western blot data (Supplementary Figure S2A), and the antibody that does not detect BCL2 in SUDHL6 also showed no off-target binding in this assay (Supplementary Figure S2A). These results indicate that our method of intracellular protein analysis is a sufficiently accurate measure of BCL2, BCLX, BCLW, and MCL1 protein levels. 

We then analyzed fresh DLBCL patient samples obtained from lymph node biopsies. There were varying levels of BCL2, BCLX, BCLW, and MCL1 in GCB and non-GCB DLBCL subtypes (Figure 2C, Supplementary Figure S2B). The levels of these proteins were typically elevated compared to their levels in normal B-cells (Figure 2D), which is consistent with the elevated RNA levels of these genes in DLBCL patient samples we previously reported [47]. For those samples with sufficient DLBCL cells, we evaluated single agents and, when possible, combination treatments with BCL2 family inhibitors. DLBCL samples showed some sensitivity to venetoclax alone (Figure 2C, Supplementary Figure S2C), and this was similar to that in normal B-cells (Figure 2D). While there were modest effects of the BCLX and the MCL1 inhibitors alone, these did not necessarily correlate to their expression level; however, combinations of these inhibitors with venetoclax showed cooperativity (Figure 2C). Notably, lower concentrations of each inhibitor used in combination with venetoclax typically resulted in better cell killing than the highest concentrations of each inhibitor alone, indicating cooperative lethal effects of the combinations. These data demonstrate possible benefits to combination treatment with venetoclax and BCLXi or MCL1i for DLCBL, but investigations for additional options are certainly warranted due to potential toxicities with these combinations.

In addition to DLBCL, we also evaluated BCL2, BCLX, BCLW, and MCL1 expression and the effects of single and combination inhibitors in patient samples of indolent lymphomas that can progress to large B-cell lymphoma [48,49], specifically follicular lymphomas that were either low- or high-grade and marginal zone lymphoma. Low-grade follicular lymphoma samples showed increased BCL2 levels and high-grade lower levels, as would be expected (Figure 2E, Supplementary Figure S2D [47]). Moreover, low-grade was more sensitive to venetoclax, navitoclax, and MCL1 inhibition compared to high-grade, and combinations of venetoclax with the other inhibitors showed more efficacy in low-grade (Figure 2E, Supplementary Figure S2E). For marginal zone lymphoma cells, levels of BCL2 family members were similar to DLBCL, but they were quite sensitive to venetoclax, navitoclax, and MCL1 inhibition, and combination treatments with venetoclax showed cooperation (Figure 2F, Supplementary Figure S2F). Together, patient samples of DLBCL, follicular lymphoma, and marginal zone lymphoma were sensitive to combination treatments of venetoclax plus BCLXi or MCL1i, showing cooperative effects with two BCL2 family inhibitors.

### 3.5. Transcriptomic and Drug Screen Analyses Identify Oxidative Phosphorylation as a Target-Able Vulnerability in Acquired Venetoclax-Resistant DLBCL

**Figure 3:**
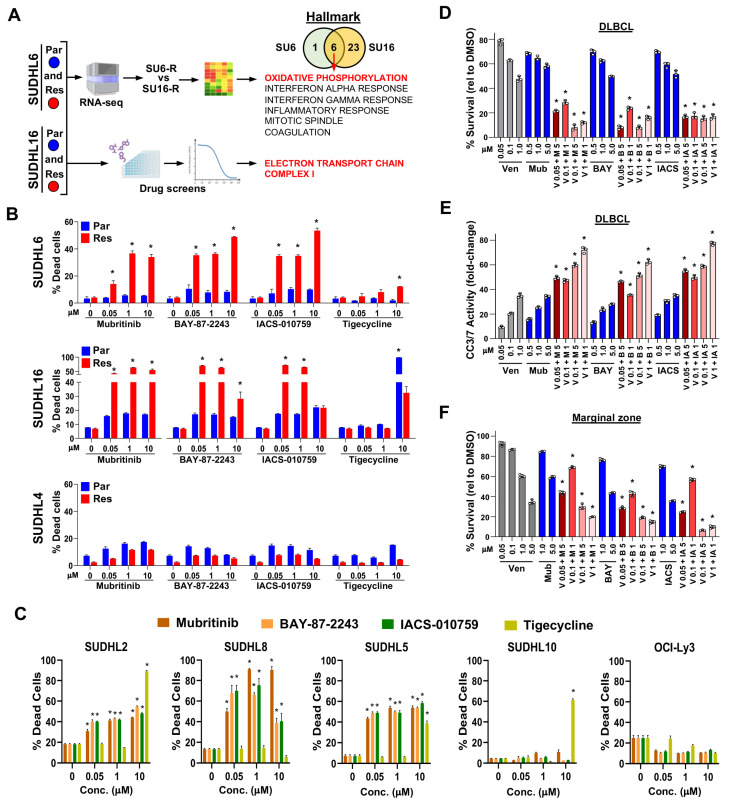
Targetable oxidative phosphorylation vulnerability identified in venetoclax-resistant DLBCL lines and lymphoma patient samples. (**A**) Schematic of the workflow of RNA-seq analysis and drug/compound screens (images modified from BioRender.com) with the cell line comparisons indicated and the number of genesets identified (Venn diagram). The six overlapping Hallmark genesets are listed. (**B**,**C**) Cell death caused by ETC inhibitor or tigecycline-treated acquired venetoclax-resistant and parental DLBCL lines (**B**) or intrinsically venetoclax-resistant DLBCL lines (**C**) was measured with live/dead flow cytometry assay (72–96 h, triplicates, relative to DMSO vehicle control, mean ± SD). For (**B**) * *p* < 0.01 (SUDHL6) and * *p* < 0.05 (SUDHL16), comparing each resistant cell line to its parental counterpart at each concentration. For (**C**) * *p* < 0.0001, comparing treated cells at each concentration to untreated cells. (**D**–**F**) Treatment of DLBCL (**D**,**E**) and marginal zone (**F**) lymphoma patient samples with venetoclax (Ven, V), mubritinib (Mub, M), BAY-87-2243 (BAY, B), and/or IACS-010759 (IACS, IA). Cell viability assays ((**D**,**F**), 24 h, triplicates, relative to DMSO vehicle control, mean ± SD) and Caspase-3/7 activity assay ((**E**), 24 h, triplicates, fold-change relative to DMSO vehicle control, mean ± SD). (**D**–**F**), * *p* < 0.0001, comparing each combination treatment to both single agent treatments at the same concentrations.

To gain insight into pathways that may confer venetoclax resistance in DLBCL cells and identify new targets to overcome or circumvent it, we performed RNA-sequencing and drug/compound screens on both the parental and acquired venetoclax-resistant SUDHL6 and SUDHL16 DLBCL lines (Figure 3A). Given that the mechanism of venetoclax resistance in SUDHL4 cells was an acquired point mutation in the BH3 domain, they were not included in these analyses. Evaluation of the RNA-sequencing data revealed six Hallmark genesets significantly increased (FDR < 0.05) in both acquired venetoclax-resistant lines compared to their respective venetoclax-sensitive parental lines, with oxidative phosphorylation a top pathway identified (Figure 3A and Supplementary Table S5). Additionally, screening of approximately 2000 compounds showed that compared to their venetoclax-sensitive parental cells, venetoclax-resistant DLBCL cells had increased sensitivity to mubritinib, initially thought to be a HER2 inhibitor [50], but subsequently shown to inhibit the mitochondrial electron transport chain (ETC; [51]) (Figure 3A, Supplementary Table S6). Oxidative phosphorylation is the engine of the ETC in mitochondria, so the overlap between both approaches revealed that the same pathway is impacted by DLBCL cells acquiring venetoclax resistance.

Since both the RNA-sequencing and drug/compound screening results suggested that the venetoclax-resistant DLBCL lines should be more sensitive to inhibitors of mitochondrial oxidative phosphorylation, we treated them with mitochondrial ETC complex I inhibitors, mubritinib, BAY-87-2243, and IACS-010759 [51,52,53], and for comparison, a mitochondrial protein translation inhibitor tigecycline [54]. Under our culture conditions, the acquired venetoclax-resistant lines SUDHL6 and SUDHL16 were both significantly more sensitive to ETC inhibition compared to their venetoclax-sensitive parental lines, showing an increase in non-viable cells (Figure 3B). In contrast, tigecycline only had an effect at a high concentration in SUDHL16 parental and resistant cells. The SUDHL4 acquired venetoclax-resistant cells were not sensitive to ETCi (Figure 3B), suggesting that the BCL2 BH3-domain mutation did not increase reliance on the ETC. Evaluation of the intrinsically venetoclax-resistant DLBCL lines showed three of the five lines had increased cell death with ETC inhibition (Figure 3C). Further evaluation of SUDHL10, which is both venetoclax-resistant and ETC inhibition-resistant, showed no detectable BCL2 protein expression (Supplementary Figure S2G). These results indicate that venetoclax-resistant DLBCL cells have an increased dependence on ETC complex I and that acquired venetoclax resistance due to *BCL2* mutation or intrinsic resistance from BCL2 expression loss does not confer this reliance. 

We next tested the efficacy of the ETC inhibitors alone and in combination with venetoclax in DLBCL patient samples. There was some sensitivity within 24 h to venetoclax and the ETC inhibitors alone, but only at high concentrations (Figure 3D). However, combination treatments with even low concentrations of venetoclax with any of the three ETC inhibitors showed significantly reduced survival over that of the compounds alone (Figure 3D). There was also significantly increased cleaved Caspase-3/7 activity with combination treatment (Figure 3E). Similarly, a marginal zone lymphoma patient sample also showed increased sensitivity to venetoclax when combined with ETC inhibition (Figure 3F). Together, these data verify the RNA-sequencing and drug/compound screening results and reveal that the ETC pathway is a targetable vulnerability in venetoclax-resistant DLBCL cells and patient samples.

### 3.6. Synergistic Effects Co-Targeting BCL2 and IDH2 in Venetoclax-Resistant DLBCL

**Figure 4:**
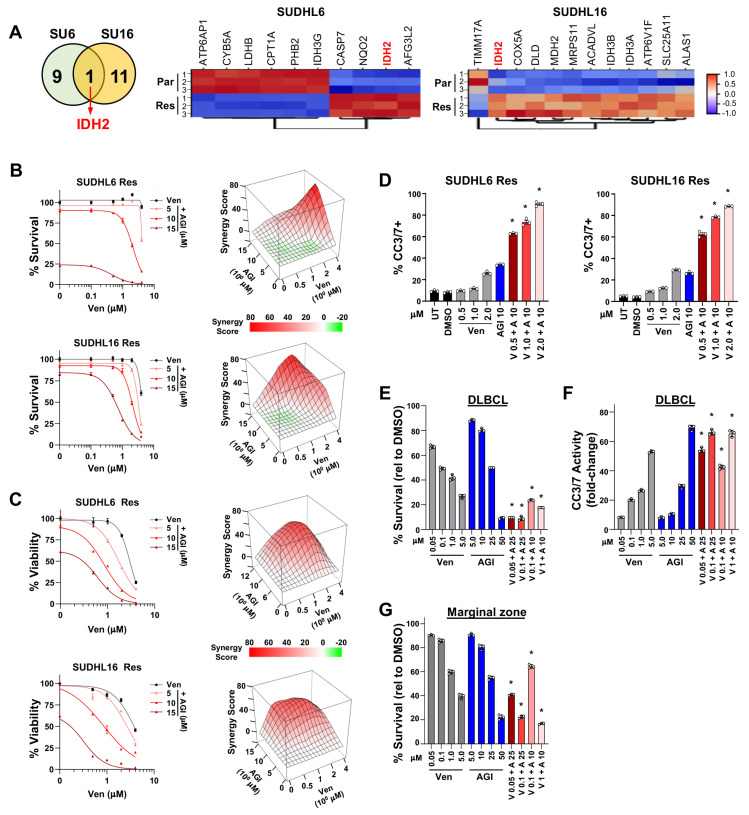
IDH2 is upregulated in acquired venetoclax-resistant DLBCL cells, and its inhibition synergizes with venetoclax to overcome resistance. (**A**) Number of overlapping and non-overlapping genes identified from Hallmark oxidative phosphorylation geneset evaluation after the comparisons shown in Figure 3A were performed. Venn diagram (left) and significantly (FDR < 0.05 with ≥1.5-fold change) altered genes are in the heatmaps (right). (**B**–**D**) Combination treatment with venetoclax (Ven, V) + IDH2 inhibitor (AGI-6780, AGI, A) in acquired venetoclax-resistant DLBCL lines. Survival assays ((**B**), left, 48 h, quadruplicates, relative to DMSO vehicle control, mean ± SEM) and 3D ZIP synergy plots ((**B**), right), live/dead flow cytometry analysis ((**C**), left, 48 h, triplicates, relative to DMSO vehicle control, mean ± SEM) and 3D ZIP synergy plots ((**C**), right), and Caspase-3/7 activity measured ((**D**), 48 h, triplicates, mean ± SD). Untreated (UT). (**E**–**G**) Treatment of DLBCL (**E**,**F**) and marginal zone lymphoma (**G**) patient samples with venetoclax (Ven, V) and AGI-6780 (AGI, A). Cell viability assays for IDH2i ((**E**,**G**), 24 h, triplicates, relative to DMSO vehicle control, mean ± SD) and Caspase-3/7 activity ((**F**), 24 h, triplicates, fold-change relative to DMSO vehicle control, mean ± SD). For (**D**–**G**) * *p* < 0.0001, comparing each combination treatment to both single-agent treatments at the same concentrations.

Further analysis of our DLBCL RNA-seq data showed that of the Hallmark oxidative phosphorylation pathway genes, *IDH2* was the only gene that showed significantly (FDR < 0.05 with 1.5-fold cutoff) increased expression in both the SUDHL6 and SUDHL16 acquired venetoclax-resistant lines compared to their parental lines (Figure 4A). IDH2 is a mitochondrial enzyme that produces NADPH to regulate redox [55]. Also, different subunits (A,B,G) of the heterotetramer IDH3 gene that regulates the TCA cycle had altered expression but did not overlap between the two cell lines. Given that *IDH2* was upregulated with acquired venetoclax resistance, we treated the SUDHL6 and SUDHL16 venetoclax-resistant lines with AGI-6780, an IDH2 inhibitor with affinity for wild-type IDH2 [56,57,58]. IDH2i alone reduced DLBCL survival of both lines only at high concentrations. However, when IDH2i was combined with venetoclax, it showed synergy with reduced DLBCL survival (Figure 4B), reduced viable cells (Figure 4C), and increased apoptosis (Figure 4D). Similarly, treatment of DLBCL patient cells had some sensitivity to single-agent treatment with venetoclax or the IDH2 inhibitor (Figure 4E). However, combination treatment with even low concentrations of venetoclax with the IDH2 inhibitor showed significantly reduced DLBCL patient cell survival and increased cleaved Caspase-3/7 activity over that of the compounds alone (Figure 4E,F). Additionally, a patient sample of marginal zone lymphoma also showed increased sensitivity to venetoclax when combined with IDH2 inhibition (Figure 4G). These results indicate that targeting IDH2 was able to overcome venetoclax resistance and was synergistic when combined with venetoclax, revealing a potential new treatment approach to overcome venetoclax resistance.

### 3.7. Drug/Compound Screens Identify Targetable Pathways to Circumvent DLBCL Venetoclax Resistance

**Figure 5:**
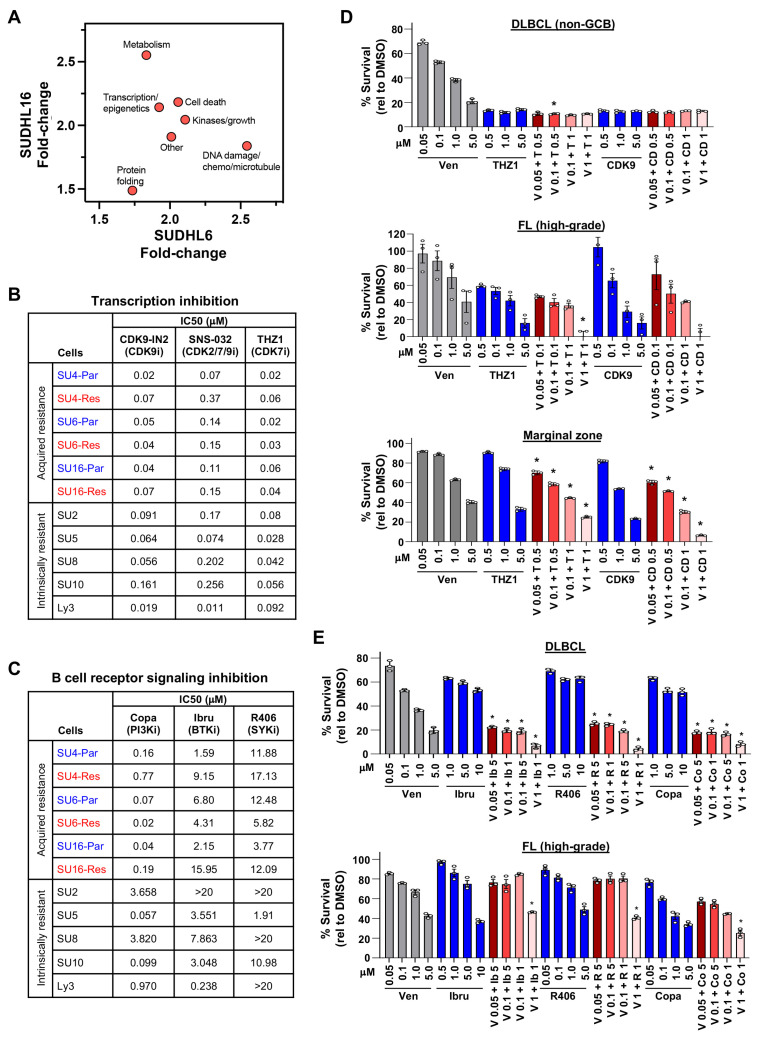
Targetable critical pathways in venetoclax-sensitive and -resistant DLBCL revealed from drug/compound screening. (**A**) Median fold-change of all compounds in the categories indicated with ≥1.5-fold increased sensitivity in acquired venetoclax-resistant DLBCL lines compared to venetoclax-sensitive lines identified by drug screens. (**B**,**C**) IC50s (μM) of acquired venetoclax-resistant (Res) and parental (Par) DLBCL lines and intrinsically venetoclax-resistant DLBCL lines following 48 h of treatment with CDK7/9 (**B**) or BCR (**C**) inhibitors. Transcription inhibitors: CDK9-IN-2 (CDK9i), SNS-032 (CDK2/7/9i), THZ1 (CDK7i). B-cell receptor signaling inhibitors: copanlisib (Copa, PI3Ki), ibrutinib (Ibru, BTKi), and R406 (SYKi). (**D**,**E**) MTS assays of enriched B-cells from fresh DLBCL (**D**,**E**), follicular (**D**,**E**), and marginal zone (**D**) lymphoma patient samples treated 24 h with venetoclax (Ven, V), THZ1 (T), CDK9 (CD), ibrutinib (Ib), R406 (R), and/or copanlisib (Copa, Co) (triplicates, relative to DMSO vehicle control, mean ± SD). For (**D**) * *p* < 0.05 (top), * *p* < 0.01 (middle), and * *p* < 0.0001 (bottom), and for (**E**) * *p* < 0.0001, comparing each combination treatment to both single-agent treatments at the same concentrations.

Ideally, drugs capable of killing both venetoclax-resistant and -sensitive DLBCL cells could be used to eliminate both cellular populations in patients. Therefore, we evaluated our drug/compound screening data further and identified five drugs/compounds that exhibited a large (>1 log) difference between the derived venetoclax-resistant lines and their venetoclax-sensitive parental lines. Three proteins targeted by these drugs/compounds were in the PI3K/AKT/mTOR pathway (Supplementary Table S6). Those drugs/compounds that showed approximately 1.5-fold or greater sensitivity in venetoclax-resistant DLBCL cells compared to their parental lines were grouped into pathway categories based on their targets (Supplementary Table S6). In addition to metabolism already described above, pathways targeting transcription/epigenetics, kinases/cell growth, DNA damage/chemo/microtubule, cell death, and protein folding showed a small increase in sensitivity in the venetoclax-resistant lines (Spearman’s rank correlation score 0.095 with *p* = 0.84; Figure 5A, Supplementary Table S6). From these data, we focused on two groups of compounds: those that target transcription/epigenetics, including CDK7/9 inhibitors that regulate RNA pol II for transcription [59,60,61] and histone deacetylases [62], and those that target kinases/growth, focusing on the B-cell receptor signaling pathway proteins (PI3K, BTK, SYK) [63] for follow-up experiments. 

Treatment of DLBCL cells with CDK7/9 inhibitors (CDK9-IN-2, SNS-032, and THZ1) showed that both SUDHL6 and SUDHL16 venetoclax-sensitive and -resistant DLBCL cells were killed by CDK7/9 inhibition at similar IC50 concentrations, whereas SUDHL4 parental cells were more sensitive than the BH3-mutant SUDHL4 resistant cells (Figure 5B, Supplementary Figure S3A). Intrinsically venetoclax-resistant lines had similar IC50 concentrations to the CDK7/9 inhibitors, with a few exceptions showing increased sensitivity (Figure 5B, Supplementary Figure S3B). We also tested class I histone deacetylase (HDAC) inhibitors (vorinostat, panobinostat, and romidepsin), which impact transcription and chromatin remodeling [62]. All three acquired venetoclax-resistant and their parental venetoclax-sensitive DLBCL lines were equally sensitive (had analogous IC50s) to HDAC inhibition, with the greatest sensitivity to romidepsin followed by panobinostat and then vorinostat (Supplementary Figure S3C). The intrinsically venetoclax-resistant lines were similarly sensitive to the HDAC inhibitors (Supplementary Figure S3D). 

Inhibition of the BCR signaling pathway had mixed results. Targeting PI3K with the pan-class I PI3K inhibitor, copanlisib, which has a greater affinity for α and δ forms, showed that the venetoclax-resistant SUDHL6 line was more sensitive than its parental line, while the parental SUDHL16 and SUDHL4 lines were more sensitive than their venetoclax-resistant counterparts (Figure 5C, Supplementary Figure S4A). Inhibition of the BCR signaling pathway kinases, BTK with ibrutinib and SYK with R406, again indicated that the venetoclax-resistant SUDHL6 cells had increased sensitivity, whereas the other two venetoclax-resistant lines were less sensitive than their parental lines (Figure 5C, Supplementary Figure S4A). All lines were least sensitive to R406 (SYKi). Four of five intrinsically venetoclax-resistant DLBCL cells showed more sensitivity to copanlisib than either ibrutinib or R406 (Figure 5C, Supplementary Figure S4B). Taken together, although drug/compound screens showed increased sensitivity of venetoclax-resistant lines compared to their venetoclax-sensitive parental lines to several pathways, our data show that there were typically analogous sensitivities with a few exceptions. However, targeting transcription initiation, transcription modulators, and the BCR signaling pathway was able to circumvent venetoclax resistance most of the time. 

### 3.8. DLBCL Patient Samples Were Sensitive to CDK7/9 and BCR Pathway Inhibitors

Next, we evaluated the efficacy of compounds that emerged from the drug screens in lymphoma patient samples in combination with venetoclax. Treatment with CDK7/9 inhibitors together with venetoclax resulted in increased DLBCL, follicular lymphoma, and marginal zone lymphoma patient sample cell death (Figure 5D). DLBCL and high-grade follicular lymphoma patient samples were also sensitive to inhibition of BCR signaling with PI3Ki, BTKi, or SYKi in combination with venetoclax (Figure 5E). High-grade follicular lymphoma also showed increased cell death with HDACi and venetoclax (Supplementary Figure S5). Therefore, the DLBCL patient sample data are consistent with the DLBCL cell line data, demonstrating that DLBCL cells are sensitive to inhibition of the multiple pathways we identified in this study and can cooperate with venetoclax. Our data also show that two additional types of B-cell lymphoma are also sensitive to these inhibitors, revealing multiple potential avenues for treating these B-cell lymphomas as well as DLBCL. Together, our data reveal critical new insights into the vulnerabilities of venetoclax resistance in B-cell lymphomas and new approaches to target these. 

## 4. Discussion

Venetoclax is FDA approved in AML and CLL with 1p deletion and in advanced clinical development in molecular subtypes of multiple myeloma and mantle cell lymphoma. However, clinical trial data in B-cell lymphoma are limited, and the low efficacy of venetoclax as a single agent in DLBCL and other B-cell lymphomas suggests that lymphoma cells are mostly resistant to BCL2 inhibition alone [20]. To date, multiple mechanisms of venetoclax resistance have been elucidated in clinical trials in AML, CLL, multiple myeloma [64], and mantle cell lymphoma [65], but no data are available for DLBCL. With multiple approaches, we have elucidated the diversity of mechanisms of venetoclax resistance already present or that developed in DLBCL, reflecting the genetic heterogeneity and clinical complexity of DLBCL. The frequent overexpression of two or more anti-apoptotic BCL2 family members in DLBCL lines and patient samples [47] prevents targeting specific BCL2 family members across all DLBCL patients. However, elevated BCL2 and BAK levels and reduced BCLW levels were a shared feature among intrinsically venetoclax-sensitive DLBCL lines, which was the opposite in the intrinsically venetoclax-resistant lines, suggesting a reciprocal relationship of these proteins and a potential for using them as biomarkers of venetoclax sensitivity/resistance. Additionally, we identified several vulnerabilities specific to venetoclax-resistant DLBCL cells, including sensitivity to inhibitors of the mitochondrial ETC complex I. Moreover, we demonstrate a novel and potentially synergistic treatment strategy for DLBCL by targeting the redox enzyme IDH2, together with venetoclax, to overcome venetoclax resistance. Furthermore, venetoclax resistance in DLBCL could be circumvented by inhibiting transcription with CDK7/9i, BCR signaling with BTKi, SYKi, or PI3Ki, and deacetylation with class I HDACi. Together, the data reveal multiple vulnerabilities and clinically viable avenues for combination treatments for venetoclax-resistant DLBCL and potentially for follicular and marginal zone lymphomas.

Overexpression of BCLX, BCLW, MCL1, and/or BFL1 causes resistance to venetoclax and navitoclax in hematologic malignancies [10,35,43,66]. In AML, CLL, and mantle cell lymphoma, upregulation and reliance on BCLX and MCL1 were reported to account for venetoclax resistance [35,42,43,67]. In DLBCL cell lines, increased AKT activation was associated with downstream upregulation of anti-apoptotic BCL2 proteins, leading to venetoclax resistance [43]. Our data show that DLBCL with acquired venetoclax resistance had increased BCLX levels, but they were not more sensitive to BCLX inhibition. Although we determined that the combination of venetoclax and an MCL1 inhibitor was synergistic and overcame venetoclax resistance in DLBCL, the clinical development of MCL1 inhibitors is impeded by cardiac toxicity, likely through an apoptosis-independent mechanism in cardiomyocytes [10,68]. 

Increased levels of any anti-apoptotic BCL2 family member could serve to sequester pro-apoptotic BH3-only BCL2 family members, such as BIM, BAX, PUMA, and/or NOXA upon BCL2 inhibition to prevent apoptosis activation [10,25]. We observed increased levels of more than one anti-apoptotic BCL2 family member in DLBCL lines, and this changed with acquired venetoclax resistance. Notably, BCLW and BFL1 are understudied BCL2 family members and only recently have been shown to contribute to DLBCL [47,66,69]. Specifically, our evaluation of 1490 DLBCL patient samples revealed significantly increased levels of *BCLW* mRNA in DLBCL, and for patients with lower levels of *BCL2*, increased *BCLW* levels correlated with reduced survival and was an independent negative prognostic indicator of survival [47,66]. Here, our data showed lower BCLW levels in venetoclax-sensitive DLBCL cells and higher levels in venetoclax-resistant DLBCL lines. For BFL1, we previously reported that there was no significant overall increase in *BFL1* mRNA in DLBCL patient samples compared to normal B-cells, but a fraction of DLBCL samples did overexpress *BFL1* [47]. Here, we show that two of the three derived venetoclax-resistant lines overexpressed the BFL1 protein. Consistent with our observation, it has been reported that compared to other hematologic malignancies, BFL1 levels are increased in DLBCL, and DLBCL lines that had increased BFL1 levels were more resistant to venetoclax [70]. Combined, our data provide additional critical evidence that changes in BCL2 family members contribute to venetoclax resistance, but it is likely also reliant on other acquired alterations during DLBCL development. 

Acquired mutation of the venetoclax-binding BH3 domain in *BCL2*, which reduces its ability to bind venetoclax, has been characterized in CLL patients and DLBCL cell lines as a mechanism of venetoclax resistance [35,37]. One of our three acquired venetoclax-resistant DLBCL lines mutated *BCL2* in the BH3-binding domain. Additionally, *BCL2* amplicon loss was detected in the HBL-2 mantle cell lymphoma line and the VAL double-hit/HGBL lymphoma line [71], although this is unlikely to be a major venetoclax resistance mechanism in DLBCL since *BCL2* amplification in DLBCL occurs infrequently [72]. Venetoclax resistance mechanisms are not limited to alterations only in *BCL2*, as acquired mutations in pro-apoptotic *BAX* have been identified in mantle cell lymphoma [36], CLL [39], and AML [38], but none of our acquired venetoclax-resistant DLBCL lines generated a *BAX* mutation. More-over, loss or mutation of *TP53* was identified as a mechanism of venetoclax resistance in AML [40,41]. However, for the DLBCL lines we tested, both venetoclax-sensitive and -resistant lines had *TP53* mutations, suggesting that *TP53* mutations may not be a significant venetoclax resistance mechanism in DLBCL, but this would need further testing. 

A reported mechanism of AML venetoclax resistance, independent of BCL2 family members, involves changes to the mitochondrial cristae structure, impacting oxidative phosphorylation [73]. Recently, two MYC/BCL2 double-hit/HGBL DLBCL cell lines were shown to be sensitive to combination treatment of tigecycline, a mitochondrial translation inhibitor, plus doxycycline and venetoclax [74], but neither cell line was sensitive to tigecycline alone, which is consistent with our findings. However, the DLBCL lines that we derived to be venetoclax-resistant and retained wild-type *BCL2* showed significantly increased sensitivity to mitochondrial ETC complex I inhibitors and an upregulation of genes in oxidative phosphorylation. Conversely, venetoclax resistance due to BCL2 BH3-domain mutation (SUDHL4) and loss of BCL2 expression (SUDHL10) was insufficient to confer increased reliance on the ETC. Moreover, three of the four intrinsically venetoclax-resistant DLBCL lines that expressed BCL2 also showed sensitivity to ETC inhibition, suggesting this is a common mechanism of venetoclax resistance in DLBCL. Of note, venetoclax-resistant DLBCL lines with a MYC translocation/amplification, only a BCL2 translocation/amplification, or lacking these showed ETC sensitivity, indicating that ETCi sensitivity did not require these genetic events. Consistent with this conclusion, it was reported that CLL and two DLBCL cell lines that were venetoclax-resistant were sensitive to ETC complex III and V inhibition [75]. Notably, we determined that IDH2 inhibition combined with venetoclax synergistically overcame DLBCL venetoclax resistance. Because IDH2 is an essential mitochondrial enzyme that produces NADPH to regulate redox, these data further reveal that mitochondrial redox is vital for venetoclax resistance. Therefore, our data in both acquired and intrinsically venetoclax-resistant DLBCL lines significantly extend and strengthen the conclusion that changes in mitochondrial oxidative phosphorylation regulate venetoclax sensitivity and highlight the critical role of the IDH2 pathway in DLBCL venetoclax resistance.

We also determined that DLBCL cells, regardless of venetoclax resistance, were sensitive to inhibitors of CDK7/9 that modulate transcription [59,60,61]. This is likely due to reliance on critical short-lived proteins, such as MYC, MCL1, and/or BFL1, and other proteins that drive growth and promote cell survival. Previously, the CDK9 inhibitor AZD4573 was shown to lead to reduced MCL1 and BFL1 levels and death of OCI-Ly10 cells and DLBCL patient-derived xenografts in vivo [70]. Venetoclax-resistant derived HBL-2 mantle cell lymphoma cells were sensitive to CDK7 inhibition with THZ1, which also prevented the emergence of venetoclax-resistant HBL-2 clones [71]. Moreover, CPI203, which inhibits BRD proteins necessary for transcription, conferred venetoclax sensitivity to double-hit/HGBL DLBCL [76]. Our data also show that inhibition of class I HDACs, which can deacetylate/upregulate genes involved in cell death, is effective at killing DLBCL cells irrespective of venetoclax resistance. Therefore, the inhibition of transcription pathways we identified can be targeted to elicit venetoclax-resistant DLBCL cell death.

## 5. Conclusions

Our findings demonstrate that multiple, complex mechanisms of venetoclax resistance can emerge in DLBCL. However, our elucidation of the increased vulnerability of venetoclax-resistant DLBCL to ETC complex I and IDH2 inhibition revealed potential new treatment approaches to overcome venetoclax resistance. Although there is still interest in adding venetoclax to decrease the threshold of apoptosis in the therapeutic armamentarium for DLBCL as a combination therapy, targeting other BCL2 family members, such as BCLW and BFL1, for which there are currently no specific targeted agents, could also be an option. It will also be important in future studies to further investigate the impact of DLBCL molecular subtype on venetoclax resistance/sensitivity, particularly due to the heterogeneity of sensitivity to different inhibitors and combinations of them that may be associated with pre-existing genetic alterations.

## Data Availability

Data are contained within the article and supplementary materials.

## Supplementary Materials

The following supporting information can be downloaded at: https://www.mdpi.com/article/10.3390/cancers16112130/s1, Figure S1: Synergy in DLBCL lines with combination treatment of venetoclax and BCLXi or MCLi; Figure S2: Intracellular levels of BCL2 family members and treatment of B-cell lymphoma patient samples with BCL2 family member inhibitors; Figure S3: CDK7/9i and HDACi circumvent venetoclax-resistant DLBCL; Figure S4: BCRi can decrease survival of venetoclax-resistant DLBCL; Figure S5: HDACi synergizes with venetoclax in follicular lymphoma. Table S1. Cell lines, reagents, and software/online tools/databases used in this study; Table S2. Patient sample information; Table S3. Inherent and acquired mutations in BCL2, BAX, and TP53 in DLBCL cell lines; Table S4. Synergy analyses for combination treatments in DLBCL lines; Table S5. Upregulated Hallmark pathways with FDR < 0.05; Table S6. Compounds identified from the drug screen with approximately 1.5-fold or more sensitivity in the venetoclax resistant line than their parental counterpart. References [77,78,79,80,81,82] are cited in the supplementary materials.

## References

[1] Liu Y., Barta S.K. (2019). Diffuse large B-cell lymphoma: 2019 update on diagnosis, risk stratification, and treatment. Am. J. Hematol..

[2] Xu-Monette Z.Y., Wu L., Visco C., Tai Y.C., Tzankov A., Liu W.M., Montes-Moreno S., Dybkaer K., Chiu A., Orazi A. (2012). Mutational profile and prognostic significance of TP53 in diffuse large B-cell lymphoma patients treated with R-CHOP: Report from an International DLBCL Rituximab-CHOP Consortium Program Study. Blood.

[3] Deng M., Xu-Monette Z.Y., Pham L.V., Wang X., Tzankov A., Fang X., Zhu F., Visco C., Bhagat G., Dybkaer K. (2021). Aggressive B-cell Lymphoma with MYC/TP53 Dual Alterations Displays Distinct Clinicopathobiological Features and Response to Novel Targeted Agents. Mol. Cancer Res. MCR.

[4] Alizadeh A.A., Eisen M.B., Davis R.E., Ma C., Lossos I.S., Rosenwald A., Boldrick J.C., Sabet H., Tran T., Yu X. (2000). Distinct types of diffuse large B-cell lymphoma identified by gene expression profiling. Nature.

[5] Rosenwald A., Wright G., Chan W.C., Connors J.M., Campo E., Fisher R.I., Gascoyne R.D., Muller-Hermelink H.K., Smeland E.B., Giltnane J.M. (2002). The use of molecular profiling to predict survival after chemotherapy for diffuse large-B-cell lymphoma. N. Engl. J. Med..

[6] Chapuy B., Stewart C., Dunford A.J., Kim J., Kamburov A., Redd R.A., Lawrence M.S., Roemer M.G.M., Li A.J., Ziepert M. (2018). Molecular subtypes of diffuse large B cell lymphoma are associated with distinct pathogenic mechanisms and outcomes. Nat. Med..

[7] Schmitz R., Wright G.W., Huang D.W., Johnson C.A., Phelan J.D., Wang J.Q., Roulland S., Kasbekar M., Young R.M., Shaffer A.L. (2018). Genetics and Pathogenesis of Diffuse Large B-Cell Lymphoma. N. Engl. J. Med..

[8] Wright G.W., Huang D.W., Phelan J.D., Coulibaly Z.A., Roulland S., Young R.M., Wang J.Q., Schmitz R., Morin R.D., Tang J. (2020). A Probabilistic Classification Tool for Genetic Subtypes of Diffuse Large B Cell Lymphoma with Therapeutic Implications. Cancer Cell.

[9] Lacy S.E., Barrans S.L., Beer P.A., Painter D., Smith A.G., Roman E., Cooke S.L., Ruiz C., Glover P., Van Hoppe S.J.L. (2020). Targeted sequencing in DLBCL, molecular subtypes, and outcomes: A Haematological Malignancy Research Network report. Blood.

[10] Adams C.M., Clark-Garvey S., Porcu P., Eischen C.M. (2018). Targeting the Bcl-2 Family in B Cell Lymphoma. Front. Oncol..

[11] Vogler M., Walter H.S., Dyer M.J.S. (2017). Targeting anti-apoptotic BCL2 family proteins in haematological malignancies—From pathogenesis to treatment. Br. J. Haematol..

[12] Huang J.Z., Sanger W.G., Greiner T.C., Staudt L.M., Weisenburger D.D., Pickering D.L., Lynch J.C., Armitage J.O., Warnke R.A., Alizadeh A.A. (2002). The t(14;18) defines a unique subset of diffuse large B-cell lymphoma with a germinal center B-cell gene expression profile. Blood.

[13] Davis R.E., Brown K.D., Siebenlist U., Staudt L.M. (2001). Constitutive nuclear factor kappaB activity is required for survival of activated B cell-like diffuse large B cell lymphoma cells. J. Exp. Med..

[14] Iqbal J., Sanger W.G., Horsman D.E., Rosenwald A., Pickering D.L., Dave B., Dave S., Xiao L., Cao K., Zhu Q. (2004). BCL2 translocation defines a unique tumor subset within the germinal center B-cell-like diffuse large B-cell lymphoma. Am. J. Pathol..

[15] Monni O., Joensuu H., Franssila K., Klefstrom J., Alitalo K., Knuutila S. (1997). BCL2 overexpression associated with chromosomal amplification in diffuse large B-cell lymphoma. Blood.

[16] Iqbal J., Meyer P.N., Smith L.M., Johnson N.A., Vose J.M., Greiner T.C., Connors J.M., Staudt L.M., Rimsza L., Jaffe E. (2011). BCL2 predicts survival in germinal center B-cell-like diffuse large B-cell lymphoma treated with CHOP-like therapy and rituximab. Clin. Cancer Res..

[17] Barrans S.L., Evans P.A., O’Connor S.J., Kendall S.J., Owen R.G., Haynes A.P., Morgan G.J., Jack A.S. (2003). The t(14;18) is associated with germinal center-derived diffuse large B-cell lymphoma and is a strong predictor of outcome. Clin. Cancer Res..

[18] Alaggio R., Amador C., Anagnostopoulos I., Attygalle A.D., Araujo I.B.O., Berti E., Bhagat G., Borges A.M., Boyer D., Calaminici M. (2022). The 5th edition of the World Health Organization Classification of Haematolymphoid Tumours: Lymphoid Neoplasms. Leukemia.

[19] Riedell P.A., Smith S.M. (2018). Double hit and double expressors in lymphoma: Definition and treatment. Cancer.

[20] Davids M.S., Roberts A.W., Seymour J.F., Pagel J.M., Kahl B.S., Wierda W.G., Puvvada S., Kipps T.J., Anderson M.A., Salem A.H. (2017). Phase I First-in-Human Study of Venetoclax in Patients With Relapsed or Refractory Non-Hodgkin Lymphoma. J. Clin. Oncol. Off. J. Am. Soc. Clin. Oncol..

[21] DiNardo C.D., Pratz K., Pullarkat V., Jonas B.A., Arellano M., Becker P.S., Frankfurt O., Konopleva M., Wei A.H., Kantarjian H.M. (2019). Venetoclax combined with decitabine or azacitidine in treatment-naive, elderly patients with acute myeloid leukemia. Blood.

[22] Wei A.H., Strickland S.A., Hou J.Z., Fiedler W., Lin T.L., Walter R.B., Enjeti A., Tiong I.S., Savona M., Lee S. (2019). Venetoclax Combined With Low-Dose Cytarabine for Previously Untreated Patients With Acute Myeloid Leukemia: Results From a Phase Ib/II Study. J. Clin. Oncol..

[23] Jain N., Keating M., Thompson P., Ferrajoli A., Burger J., Borthakur G., Takahashi K., Estrov Z., Fowler N., Kadia T. (2019). Ibrutinib and Venetoclax for First-Line Treatment of CLL. N. Engl. J. Med..

[24] Morschhauser F., Feugier P., Flinn I.W., Gasiorowski R., Greil R., Illes A., Johnson N.A., Larouche J.F., Lugtenburg P.J., Patti C. (2021). A phase 2 study of venetoclax plus R-CHOP as first-line treatment for patients with diffuse large B-cell lymphoma. Blood.

[25] Diepstraten S.T., Anderson M.A., Czabotar P.E., Lessene G., Strasser A., Kelly G.L. (2022). The manipulation of apoptosis for cancer therapy using BH3-mimetic drugs. Nat. Rev. Cancer.

[26] Condoluci A., Rossi D. (2022). Mechanisms of resistance to venetoclax. Blood.

[27] Thomalla D., Beckmann L., Grimm C., Oliverio M., Meder L., Herling C.D., Nieper P., Feldmann T., Merkel O., Lorsy E. (2022). Deregulation and epigenetic modification of BCL2-family genes cause resistance to venetoclax in hematologic malignancies. Blood.

[28] Thijssen R., Tian L., Anderson M.A., Flensburg C., Jarratt A., Garnham A.L., Jabbari J.S., Peng H., Lew T.E., Teh C.E. (2022). Single-cell multiomics reveal the scale of multilayered adaptations enabling CLL relapse during venetoclax therapy. Blood.

[29] Zheng S., Wang W., Aldahdooh J., Malyutina A., Shadbahr T., Tanoli Z., Pessia A., Tang J. (2022). SynergyFinder Plus: Toward Better Interpretation and Annotation of Drug Combination Screening Datasets. Genom. Proteom. Bioinform..

[30] Adams C.M., Mitra R., Xiao Y., Michener P., Palazzo J., Chao A., Gour J., Cassel J., Salvino J.M., Eischen C.M. (2023). Targeted MDM2 Degradation Reveals a New Vulnerability for p53-Inactivated Triple-Negative Breast Cancer. Cancer Discov..

[31] Pham L.V., Huang S., Zhang H., Zhang J., Bell T., Zhou S., Pogue E., Ding Z., Lam L., Westin J. (2018). Strategic Therapeutic Targeting to Overcome Venetoclax Resistance in Aggressive B-cell Lymphomas. Clin. Cancer Res..

[32] Phillips D.C., Xiao Y., Lam L.T., Litvinovich E., Roberts-Rapp L., Souers A.J., Leverson J.D. (2015). Loss in MCL-1 function sensitizes non-Hodgkin’s lymphoma cell lines to the BCL-2-selective inhibitor venetoclax (ABT-199). Blood Cancer J..

[33] Singh G., Guibao C.D., Seetharaman J., Aggarwal A., Grace C.R., McNamara D.E., Vaithiyalingam S., Waddell M.B., Moldoveanu T. (2022). Structural basis of BAK activation in mitochondrial apoptosis initiation. Nat. Commun..

[34] Drexler H.G., Eberth S., Nagel S., MacLeod R.A. (2016). Malignant hematopoietic cell lines: In vitro models for double-hit B-cell lymphomas. Leuk. Lymphoma.

[35] Tahir S.K., Smith M.L., Hessler P., Rapp L.R., Idler K.B., Park C.H., Leverson J.D., Lam L.T. (2017). Potential mechanisms of resistance to venetoclax and strategies to circumvent it. BMC Cancer.

[36] Fresquet V., Rieger M., Carolis C., Garcia-Barchino M.J., Martinez-Climent J.A. (2014). Acquired mutations in BCL2 family proteins conferring resistance to the BH3 mimetic ABT-199 in lymphoma. Blood.

[37] Blombery P., Anderson M.A., Gong J.N., Thijssen R., Birkinshaw R.W., Thompson E.R., Teh C.E., Nguyen T., Xu Z., Flensburg C. (2019). Acquisition of the Recurrent Gly101Val Mutation in BCL2 Confers Resistance to Venetoclax in Patients with Progressive Chronic Lymphocytic Leukemia. Cancer Discov..

[38] Moujalled D.M., Brown F.C., Chua C.C., Dengler M.A., Pomilio G., Anstee N.S., Litalien V., Thompson E.R., Morley T.D., MacRaild S. (2022). Acquired mutations in BAX confer resistance to BH3-mimetic therapy in Acute Myeloid Leukemia. Blood.

[39] Blombery P., Lew T.E., Dengler M.A., Thompson E.R., Lin V.S., Chen X., Nguyen T., Panigrahi A., Handunnetti S.M., Carney D.A. (2022). Clonal hematopoiesis, myeloid disorders and BAX-mutated myelopoiesis in patients receiving venetoclax for CLL. Blood.

[40] Nechiporuk T., Kurtz S.E., Nikolova O., Liu T., Jones C.L., D’Alessandro A., Culp-Hill R., d’Almeida A., Joshi S.K., Rosenberg M. (2019). The TP53 Apoptotic Network Is a Primary Mediator of Resistance to BCL2 Inhibition in AML Cells. Cancer Discov..

[41] Thijssen R., Diepstraten S.T., Moujalled D.M., Chew E., Flensburg C., Shi M.X., Dengler M.A., Litalien V., MacRaild S., Chen M. (2021). Intact TP53 function is essential for sustaining durable responses to BH3-mimetic drugs in leukemias. Blood.

[42] Niu X., Zhao J., Ma J., Xie C., Edwards H., Wang G., Caldwell J.T., Xiang S., Zhang X., Chu R. (2016). Binding of Released Bim to Mcl-1 is a Mechanism of Intrinsic Resistance to ABT-199 which can be Overcome by Combination with Daunorubicin or Cytarabine in AML Cells. Clin. Cancer Res..

[43] Choudhary G.S., Al-Harbi S., Mazumder S., Hill B.T., Smith M.R., Bodo J., Hsi E.D., Almasan A. (2015). MCL-1 and BCL-xL-dependent resistance to the BCL-2 inhibitor ABT-199 can be overcome by preventing PI3K/AKT/mTOR activation in lymphoid malignancies. Cell Death Dis..

[44] Leverson J.D., Phillips D.C., Mitten M.J., Boghaert E.R., Diaz D., Tahir S.K., Belmont L.D., Nimmer P., Xiao Y., Ma X.M. (2015). Exploiting selective BCL-2 family inhibitors to dissect cell survival dependencies and define improved strategies for cancer therapy. Sci. Transl. Med..

[45] Szlavik Z., Ondi L., Csekei M., Paczal A., Szabo Z.B., Radics G., Murray J., Davidson J., Chen I., Davis B. (2019). Structure-Guided Discovery of a Selective Mcl-1 Inhibitor with Cellular Activity. J. Med. Chem..

[46] Merino D., Khaw S.L., Glaser S.P., Anderson D.J., Belmont L.D., Wong C., Yue P., Robati M., Phipson B., Fairlie W.D. (2012). Bcl-2, Bcl-x(L), and Bcl-w are not equivalent targets of ABT-737 and navitoclax (ABT-263) in lymphoid and leukemic cells. Blood.

[47] Adams C.M., Mitra R., Gong J.Z., Eischen C.M. (2017). Non-Hodgkin and Hodgkin Lymphomas Select for Overexpression of BCLW. Clin. Cancer Res..

[48] Lossos I.S., Gascoyne R.D. (2011). Transformation of follicular lymphoma. Best. Pract. Res. Clin. Haematol..

[49] Casulo C., Friedberg J. (2017). Transformation of marginal zone lymphoma (and association with other lymphomas). Best. Pract. Res. Clin. Haematol..

[50] Nagasawa J., Mizokami A., Koshida K., Yoshida S., Naito K., Namiki M. (2006). Novel HER2 selective tyrosine kinase inhibitor, TAK-165, inhibits bladder, kidney and androgen-independent prostate cancer in vitro and in vivo. Int. J. Urol..

[51] Baccelli I., Gareau Y., Lehnertz B., Gingras S., Spinella J.F., Corneau S., Mayotte N., Girard S., Frechette M., Blouin-Chagnon V. (2019). Mubritinib Targets the Electron Transport Chain Complex I and Reveals the Landscape of OXPHOS Dependency in Acute Myeloid Leukemia. Cancer Cell.

[52] Ellinghaus P., Heisler I., Unterschemmann K., Haerter M., Beck H., Greschat S., Ehrmann A., Summer H., Flamme I., Oehme F. (2013). BAY 87-2243, a highly potent and selective inhibitor of hypoxia-induced gene activation has antitumor activities by inhibition of mitochondrial complex I. Cancer Med..

[53] Molina J.R., Sun Y., Protopopova M., Gera S., Bandi M., Bristow C., McAfoos T., Morlacchi P., Ackroyd J., Agip A.A. (2018). An inhibitor of oxidative phosphorylation exploits cancer vulnerability. Nat. Med..

[54] Skrtic M., Sriskanthadevan S., Jhas B., Gebbia M., Wang X., Wang Z., Hurren R., Jitkova Y., Gronda M., Maclean N. (2011). Inhibition of mitochondrial translation as a therapeutic strategy for human acute myeloid leukemia. Cancer Cell.

[55] Guo J., Zhang R., Yang Z., Duan Z., Yin D., Zhou Y. (2021). Biological Roles and Therapeutic Applications of IDH2 Mutations in Human Cancer. Front. Oncol..

[56] Bergaggio E., Riganti C., Garaffo G., Vitale N., Mereu E., Bandini C., Pellegrino E., Pullano V., Omede P., Todoerti K. (2019). IDH2 inhibition enhances proteasome inhibitor responsiveness in hematological malignancies. Blood.

[57] Li J., He Y., Tan Z., Lu J., Li L., Song X., Shi F., Xie L., You S., Luo X. (2018). Wild-type IDH2 promotes the Warburg effect and tumor growth through HIF1alpha in lung cancer. Theranostics.

[58] Zeng P., Lu W., Tian J., Qiao S., Li J., Glorieux C., Wen S., Zhang H., Li Y., Huang P. (2022). Reductive TCA cycle catalyzed by wild-type IDH2 promotes acute myeloid leukemia and is a metabolic vulnerability for potential targeted therapy. J. Hematol. Oncol..

[59] Chipumuro E., Marco E., Christensen C.L., Kwiatkowski N., Zhang T., Hatheway C.M., Abraham B.J., Sharma B., Yeung C., Altabef A. (2014). CDK7 inhibition suppresses super-enhancer-linked oncogenic transcription in MYCN-driven cancer. Cell.

[60] Kwiatkowski N., Zhang T., Rahl P.B., Abraham B.J., Reddy J., Ficarro S.B., Dastur A., Amzallag A., Ramaswamy S., Tesar B. (2014). Targeting transcription regulation in cancer with a covalent CDK7 inhibitor. Nature.

[61] Bacon C.W., D’Orso I. (2019). CDK9: A signaling hub for transcriptional control. Transcription.

[62] Milazzo G., Mercatelli D., Di Muzio G., Triboli L., De Rosa P., Perini G., Giorgi F.M. (2020). Histone Deacetylases (HDACs): Evolution, Specificity, Role in Transcriptional Complexes, and Pharmacological Actionability. Genes.

[63] Burger J.A., Wiestner A. (2018). Targeting B cell receptor signalling in cancer: Preclinical and clinical advances. Nat. Rev. Cancer.

[64] Lasica M., Anderson M.A. (2021). Review of Venetoclax in CLL, AML and Multiple Myeloma. J. Pers. Med..

[65] Zhao S., Kanagal-Shamanna R., Navsaria L., Ok C.Y., Zhang S., Nomie K., Han G., Hao D., Hill H.A., Jiang C. (2020). Efficacy of venetoclax in high risk relapsed mantle cell lymphoma (MCL)—Outcomes and mutation profile from venetoclax resistant MCL patients. Am. J. Hematol..

[66] Adams C.M., Kim A.S., Mitra R., Choi J.K., Gong J.Z., Eischen C.M. (2017). BCL-W has a fundamental role in B cell survival and lymphomagenesis. J. Clin. Invest..

[67] Thus Y.J., Eldering E., Kater A.P., Spaargaren M. (2022). Tipping the balance: Toward rational combination therapies to overcome venetoclax resistance in mantle cell lymphoma. Leukemia.

[68] Yuda J., Will C., Phillips D.C., Abraham L., Alvey C., Avigdor A., Buck W., Besenhofer L., Boghaert E., Cheng D. (2023). Selective MCL-1 inhibitor ABBV-467 is efficacious in tumor models but is associated with cardiac troponin increases in patients. Commun. Med..

[69] Wang G., Diepstraten S.T., Herold M.J. (2022). Last but not least: BFL-1 as an emerging target for anti-cancer therapies. Biochem. Soc. Trans..

[70] Boiko S., Proia T., San Martin M., Gregory G.P., Wu M.M., Aryal N., Hattersley M., Shao W., Saeh J.C., Fawell S.E. (2021). Targeting Bfl-1 via acute CDK9 inhibition overcomes intrinsic BH3-mimetic resistance in lymphomas. Blood.

[71] Zhao X., Ren Y., Lawlor M., Shah B.D., Park P.M.C., Lwin T., Wang X., Liu K., Wang M., Gao J. (2019). BCL2 Amplicon Loss and Transcriptional Remodeling Drives ABT-199 Resistance in B Cell Lymphoma Models. Cancer Cell.

[72] Consortium A.P.G. (2017). AACR Project GENIE: Powering Precision Medicine through an International Consortium. Cancer Discov..

[73] Chen X., Glytsou C., Zhou H., Narang S., Reyna D.E., Lopez A., Sakellaropoulos T., Gong Y., Kloetgen A., Yap Y.S. (2019). Targeting Mitochondrial Structure Sensitizes Acute Myeloid Leukemia to Venetoclax Treatment. Cancer Discov..

[74] Rava M., D’Andrea A., Nicoli P., Gritti I., Donati G., Doni M., Giorgio M., Olivero D., Amati B. (2018). Therapeutic synergy between tigecycline and venetoclax in a preclinical model of MYC/BCL2 double-hit B cell lymphoma. Sci. Transl. Med..

[75] Guieze R., Liu V.M., Rosebrock D., Jourdain A.A., Hernandez-Sanchez M., Martinez Zurita A., Sun J., Ten Hacken E., Baranowski K., Thompson P.A. (2019). Mitochondrial Reprogramming Underlies Resistance to BCL-2 Inhibition in Lymphoid Malignancies. Cancer Cell.

[76] Esteve-Arenys A., Valero J.G., Chamorro-Jorganes A., Gonzalez D., Rodriguez V., Dlouhy I., Salaverria I., Campo E., Colomer D., Martinez A. (2018). The BET bromodomain inhibitor CPI203 overcomes resistance to ABT-199 (venetoclax) by downregulation of BFL-1/A1 in in vitro and in vivo models of MYC+/BCL2+ double hit lymphoma. Oncogene.

[77] Pertea M., Kim D., Pertea G.M., Leek J.T., Salzberg S.L. (2016). Transcript-level expression analysis of RNA-seq experiments with HISAT, StringTie and Ballgown. Nat. Protoc..

[78] Robinson M.D., McCarthy D.J., Smyth G.K. (2010). edgeR: A Bioconductor package for differential expression analysis of digital gene expression data. Bioinformatics.

[79] Benjamini Y. Y. (1995). H. Controlling the False Discovery Rate-a Practical and Powerful Approach to Multiple Testing. J. R. Stat. Soc. Ser. BMethodological.

[80] Subramanian A., Tamayo P., Mootha V.K., Mukherjee S., Ebert B.L., Gillette M.A., Paulovich A., Pomeroy S.L., Golub T.R., Lander E.S. (2005). Gene set enrichment analysis: A knowledge-based approach for interpreting genome-wide expression profiles. Proc. Natl. Acad. Sci. USA.

[81] Liberzon A., Birger C., Thorvaldsdottir H., Ghandi M., Mesirov J.P., Tamayo P. (2015). The Molecular Signatures Database (MSigDB) hallmark gene set collection. Cell Syst..

[82] Galili T., O’Callaghan A., Sidi J., Sievert C. (2018). heatmaply: An R package for creating interactive cluster heatmaps for online publishing. Bioinformatics.

