# Supplementary material for: Identifying Targetable Vulnerabilities to Circumvent or Overcome Venetoclax Resistance in Diffuse Large B-Cell Lymphoma
Source: Cancers (Basel). 2024 Jun 3;16(11):2130. doi: 10.3390/cancers16112130 (PMC11171410; doi:10.3390/cancers16112130)
Supplement: Supplementary file 1 [file cancers-16-02130-s001.zip › Adams et al DLBCL Suppl text & tables Final.pdf]

# SUPPLEMENTARY MATERIAL

## Supplementary Methods

### *RNA-sequencing*

Quality of the sequencing reads were determined with FASTQC (https://www.bioinformatics.babraham.ac.uk/projects/fastqc) and adapter sequences trimmed with TrimGalore (https://www.bioinformatics.babraham.ac.uk/projects/trim galore/). RNA-sequencing reads were aligned to GRCh38 with HISAT2 (version 2.2.1) and transcript assembling was performed with StringTie (version 2.2.1) (1). Differential gene expression analysis using the R packages edgeR (2) was done and statistical significance determined based on a *P*-value cutoff of 0.05 after being adjusted by Benjamini-Hochberg multiple test correction method (3). The Benjamini-Hochberg multiple test correction method was implemented in the edgeR tool (version 3.38.4) and applied to the nominal p-values to control the false discovery rate. We performed Gene Set Enrichment Analysis (GSEA) (4) using the cancer Hallmark dataset in the mSigDB database (version 7.5.1) (5). The heatmaps were generated using the R package heatmaply (6).

### *Drug/compound screen*

Cells (500 per/well) in 25μL of complete media were placed into white, opaque tissue culture treated 384-well plates. Compounds (SelleckChem and MedChemExpress anti-cancer libraries) were dissolved in 100% DMSO and 25nL of 1000X final concentration and dispensed using either the Janus MDT or the Echo 650 acoustic liquid handler. After a 72hr incubation (37°C, 5% $CO_2$), cell viability was determined by adding 12μL of CellTiter-Glo Luminescent Viability Assay (Promega) and luminescence measured using the ClarioStar Plus plate reader (BMG LabTech). Luminescence values were expressed as % toxicity where 0% is the luminescence in the DMSO control, and 100% is the luminescence in the presence of 10μM bortezomib.

## Supplementary References


1. Pertea M, Kim D, Pertea GM, Leek JT, Salzberg SL. Transcript-level expression analysis of RNA-seq experiments with HISAT, StringTie and Ballgown. Nat Protoc **2016**;11:1650-67
2. Robinson MD, McCarthy DJ, Smyth GK. edgeR: a Bioconductor package for differential expression analysis of digital gene expression data. Bioinformatics **2010**;26:139-40
3. Benjamini Y., Y. H. Controlling the False Discovery Rate - a Practical and Powerful Approach to Multiple Testing. Journal of the Royal Statistical Society Series B-Methodological **1995**;57:289-300
4. Subramanian A, Tamayo P, Mootha VK, Mukherjee S, Ebert BL, Gillette MA*, et al.* Gene set enrichment analysis: a knowledge-based approach for interpreting genome-wide expression profiles. Proc Natl Acad Sci U S A **2005**;102:15545-50
5. Liberzon A, Birger C, Thorvaldsdottir H, Ghandi M, Mesirov JP, Tamayo P. The Molecular Signatures Database (MSigDB) hallmark gene set collection. Cell Syst **2015**;1:417-25
6. Galili T, O'Callaghan A, Sidi J, Sievert C. heatmaply: an R package for creating interactive cluster heatmaps for online publishing. Bioinformatics **2018**;34:1600-2

## Supplementary Tables:

**Supplementary Table S1. Cell lines, reagents, and software/online tools/databases used in this study.**

| DLBCL cell lines | | | | | | | |
|---|---|---|---|---|---|---|---|
| **Cell line** | **Source** | **Subtype** | **Catalog #** | **RRID** | | | |
| SUDHL2 | ATCC | ABC | CRL-2956 | CVCL_9550 | | | |
| SUDHL4 | ATCC | GCB | CRL-2957 | CVCL_0539 | | | |
| SUDHL5 | ATCC | GCB | CRL-2958 | CVCL_1735 | | | |
| SUDHL6 | ATCC | GCB | CRL-2959 | CVCL_2206 | | | |
| SUDHL8 | ATCC | GCB | CRL-2961 | CVCL_2207 | | | |
| SUDHL10 | ATCC | GCB | CRL-2963 | CVCL_1889 | | | |
| SUDHL16 | ATCC | GCB | CRL-2964 | CVCL_1890 | | | |
| Toledo | ATCC | GCB | CRL-2631 | CVCL_3611 | | | |
| OCI-Ly3 | Dr. John Chan | ABC | n/a | CVCL_8800 | | | |
| OCI-Ly10 | Dr. John Chan | ABC | n/a | CVCL_8795 | | | |
| **Antibodies** | | | | | | | |
| **Target** | **Conjugation** | **Clone** | **Species** | **Company** | **Catalog #** | **Purpose** | **RRID** |
| BCL2 | Alexa 488 | 124 | Mouse | Cell Signaling | 59422 | IC | AB_2799566 |
| BCLX | PE-Cy7 | 54H6 | Rabbit | Cell Signaling | 81965 | IC | AB_2936839 |
| BCLX | Alexa 488 | 54H6 | Rabbit | Cell Signaling | 2767 | IC | AB_2274763 |
| BCLW* | PE | 31H4 | Rabbit | Cell Signaling | Q13405 | IC | n/a |
| MCL1 | Alexa 647 | D2W9E | Rabbit | Cell Signaling | 78471 | IC | AB_2799914 |
| Isotype | Alexa 488 | MOPC-21 | Mouse | BD Bioscience | 557721 | IC | AB_396830 |
| Isotype | PE-Cy7 | DA1E | Rabbit | Cell Signaling | 97492 | IC | n/a |
| Isotype | PE | DA1E | Rabbit | Cell Signaling | 5742 | IC | AB_10694219 |
| Isotype | Alexa 647 | DA1E | Rabbit | Cell Signaling | 2985 | IC | AB_1196589 |
| CD20 | BV786 | 2H7 | Mouse | BD Bioscience | 743611 | Surface | AB_2741622 |
| CD20 | PerCP-Cy5.5 | 2H7 | Mouse | BD Bioscience | 560736 | Surface | AB_1727451 |
| CD19 | APC-Cy7 | SJ25C1 | Mouse | BD Bioscience | 557791 | Surface | AB_396873 |
| CD19 | BV421 | HIB19 | Mouse | BD Bioscience | 562440 | Surface | AB_11153299 |
| CD19 | BV605 | SJ25C1 | Mouse | BD Bioscience | 562654 | Surface | AB_2909453 |
| CD3 | BV786 | UCHT1 | Mouse | BD Bioscience | 565491 | Surface | AB_2739260 |
| Lambda | BV421 | JDC-12 | Mouse | BD Bioscience | 562893 | Surface | AB_2737872 |
| Lambda | BV605 | JDC-12 | Mouse | BD Bioscience | 563292 | Surface | AB_2738121 |
| Kappa | BV421 | G20-193 | Mouse | BD Bioscience | 562619 | Surface | AB_2737682 |
| Kappa | BV605 | TB28-2 | Mouse | BioLegend | 392715 | Surface | AB_3068044 |
| Isotype | BV786 | X40 | Mouse | BD Bioscience | 563330 | Surface | AB_2869484 |
| Isotype | PerCP-Cy5.5 | MOPC-21 | Mouse | BD Bioscience | 550795 | Surface | AB_393885 |
| Isotype | APC-Cy7 | MOPC-21 | Mouse | BD Bioscience | 557873 | Surface | AB_396915 |
| Isotype | BV421 | X40 | Mouse | BD Bioscience | 562438 | Surface | AB_11207319 |
| Isotype | BV605 | X40 | Mouse | BD Bioscience | 562652 | Surface | AB_2714005 |
| Viability stain | BV440 | n/a | n/a | BD Bioscience | 566332 | n/a | AB_2869748 |
| BCL2 (Ab1) | n/a | 6C8 | Hamster | BD Bioscience | 551051 | WB | AB_394018 |
| BCL2 (Ab2) | n/a | 124 | Mouse | Cell Signaling | 15071 | WB | AB_2744528 |
| BCLX | n/a | 2H12 | Mouse | BD Bioscience | 551020 | WB | AB_394006 |
| BCLW | n/a | 31H4 | Rabbit | Cell Signaling | 2724 | WB | AB_10691557 |
| MCL1 | n/a | D35A5 | Rabbit | Cell Signaling | 5453 | WB | AB_10694494 |
| BFL1 | n/a | polyclonal | Rabbit | Abcam | ab45413 | WB | AB_722510 |
| BAX | n/a | N-20 | Rabbit | Santa Cruz | sc-493 | WB | AB_2227995 |
| BAK | n/a | AT38E2 | Mouse | Santa Cruz | sc-517390 | WB | n/a |
| BAD | n/a | C-7 | Mouse | Santa Cruz | sc-8044 | WB | AB_626717 |
| BID | n/a | 7 | Rabbit | BD Bioscience | 550365 | WB | AB_393636 |
| BIM | n/a | 2933 | Rabbit | Cell Signaling | 2933 | WB | AB_1030947 |

| | | | | | | | |
|---|---|---|---|---|---|---|---|
| NOXA | n/a | D8L7U | Rabbit | Cell Signaling | 14766 | WB | AB_2798602 |
| PUMA | n/a | E2P7G | Rabbit | Cell Signaling | 98672 | WB | n/a |
| MYC | n/a | Y69 | Rabbit | Abcam | ab32072 | WB | AB_731658 |
| β-ACTIN | n/a | AC-15 | Mouse | Sigma Aldrich | A5441 | WB | AB_476744 |
| Mouse-HRP | n/a | n/a | Sheep | Millipore Sigma | NA931 | WB | AB_772210 |
| Rabbit-HRP | n/a | n/a | Donkey | Millipore Sigma | NA934 | WB | AB_772206 |
| Hamster-HRP | n/a | n/a | Goat | Jackson Immuno-Research Labs | 127-035-099 | WB | AB_2338975 |
| **Compounds/inhibitors** | | | | | | | |
| **Name** | **Target** | | **Company** | | **Catalog#** | | |
| A-1331852 | BCLX | | AbbVie and SelleckChem | | n/a | | |
| AGI-6780 | IDH2 | | MedChem Express | | HY-15734 | | |
| BAY 87-2243 | ETC complex I | | MedChem Express | | HY-15836 | | |
| CDK9-IN-2 | CDK9 | | MedChem Express | | HY-16462 | | |
| Copanlisib | PI3K | | MedChem Express | | HY-15346 | | |
| IACS-010759 | ETC complex I | | MedChem Express | | HY-112037 | | |
| Ibrutinib | BTK | | MedChem Express | | HY-10997 | | |
| MIK665 | MCL1 | | MedChem Express | | HY-112218 | | |
| Mubritinib | ETC complex I | | SelleckChem | | S2216 | | |
| Navitoclax | BCL2/BCLX/BCLW | | AbbVie and SelleckChem | | n/a | | |
| Panobinostat | Class I HDAC | | MedChem Express | | HY-10224 | | |
| R406 | SYK | | MedChem Express | | HY-12067 | | |
| Romidepsin | Class I HDAC | | MedChem Express | | HY-15149 | | |
| SNS-032 | CDK2/7/9 | | MedChem Express | | HY-10008 | | |
| THZ1 | CDK7 | | MedChem Express | | HY-80013 | | |
| Tigecycline | Mitochondrial translation | | MedChem Express | | HY-B0117 | | |
| Venetoclax | BCL2 | | AbbVie and SelleckChem | | n/a | | |
| Vorinostat | Class I HDAC | | MedChem Express | | HY-10221 | | |
| **Kits/misc. reagents** | | | | | | | |
| **Kit/reagent** | | | | **Company** | **Catalog#** | | |
| CellTiter-Glo Luminescent Viability Assay | | | | Promega | G7570 | | |
| CellTiter-96 Aqueous One Solution Cell Proliferation Assay | | | | Promega | G3580 | | |
| CellTiter-Fluor Cell Viability Assay | | | | Promega | G6080 | | |
| CellEvent Caspase-3/7 Green Flow Cytometry Assay Kit | | | | Invitrogen | C10740 | | |
| CellEvent Caspase-3/7 Green Detection Reagent | | | | Invitrogen | C10423 | | |
| Live and Dead Cell Assay | | | | Abcam | ab115347 | | |
| BD Horizon Brilliant Stain Buffer | | | | BD Biosciences | 563794 | | |
| Human B cell Isolation Kit II | | | | Miltenyi Biotec | 130-091-151 | | |
| MycoSensor PCR Assay Kit | | | | Agilent Tech. | 302108 | | |
| SuperScript III First-strand Synthesis System | | | | Invitrogen | 18080051 | | |
| **Software/Online Tool/Database** | | | | | | | |
| **Name** | | | | **Type** | **RRID** | | |
| IDBS | | | | Online tool | SCR_004077 | | |
| SynergyFinderPlus | | | | Online tool | SCR_019318 | | |
| FlowJo (version 10.8.0) | | | | Software | SCR_008520 | | |
| FASTQC (version 0.12.1) | | | | Online tool | SCR_014583 | | |
| TrimGalore (version 0.6.10) | | | | Online tool | SCR_011847 | | |
| HISAT2 (version 2.2.1) | | | | Online tool | SCR_015530 | | |
| StringTie (version 2.2.1) | | | | Online tool | SCR_016323 | | |
| EdgeR (version 3.38.4) | | | | Online tool | SCR_012802 | | |
| mSigDB (version 7.5.1) | | | | Database | SCR_016863 | | |
| GraphPad Prism (10.0.0) | | | | Software | SCR_002798 | | |
| FinchTV DNA Chromatogram Viewer (version 1.4.0) | | | | Software | SCR_005584 | | |

*custom-made antibody; not applicable (n/a); intracellular (IC); Western blot (WB)

**Supplementary Table S2. Patient sample information.**

| Patient sample ID | Gender | Age | Race (Ethnicity) | Untreated or Relapsed | Diagnosis | BCL2 and/or MYC alterations | Figure sample was used |
|---|---|---|---|---|---|---|---|
| 222 | Female | 56 | White/Caucasian | Diagnostic | DLBCL, non-GCB | IHC: BCL2+<br>FISH: BCL2 amplified (3 copies) | Fig S2B |
| 223 | Female | 21 | White/Caucasian (Hispanic/Latino) | Diagnostic | DLBCL, GCB, double expressor | IHC: BCL2+, MYC+<br>FISH: MYC rearrangement | Fig S2B |
| 232 | Male | 80 | White/Caucasian | Relapse | DLBCL, non-GCB | FISH: BCL2 rearrangement# | Fig S2B |
| 243 | Male | 66 | Black/African American | Diagnostic | DLBCL, non-GCB | IHC: BCL2+<br>FISH: BCL2 amplified (4-5 copies) | Fig 2C |
| 289 | Female | 35 | Not Reported | Relapse | DLBCL, non-GCB | IHC: BCL2+<br>FISH: BCL2 amplified (5-10 copies) | Fig 3D, 3E, 4E, 4F, 5D, 5E, S2C |
| 233 | Female | 77 | White/Caucasian | Diagnostic | FL, low-grade | IHC: BCL2+<br>FISH: BCL2 rearrangement t(14;18) | Fig 2E |
| 252 | Female | 58 | White/Caucasian | Diagnostic | FL, high-grade | IHC: BCL2+ | Fig S2E |
| 255 | Male | 77 | White/Caucasian | Diagnostic | FL, low-grade | IHC: BCL2+<br>FISH: BCL2 rearrangement t(14;18) + one extra copy of fusion and one extra copy of BCL2 | Fig S2D, S2E |
| 283 | Female | 70 | Black/African American | Diagnostic | FL, high-grade | FISH: BCL2 rearrangement# | Fig 2E, 5D, 5E, S2E, S5 |
| 229 | Male | 67 | White/Caucasian | Diagnostic | MZL | IHC: BCL2+ | Fig 2F, S2F |
| 286 | Female | 57 | White/Caucasian | Relapse | MZL | IHC: BCL2+, MYC heterogenous | Fig 3F, 4G, 5D, S2F |
| 256 | Female | 67 | White/Caucasian | Diagnostic | Benign lymph node | not applicable | Fig 2D |

Immunohistochemistry (IHC); Fluorescence *In Situ* Hybridization (FISH); diffuse large B-Cell lymphoma (DLBCL); follicular lymphoma (FL); marginal zone lymphoma (MZL); BCL2+ is typically in all the cells; MYC+ is defined as >40%; #breakpoint probe used so rearrangement partner not determined

**Supplementary Table S3. Inherent and acquired mutations in *BCL2*, *BAX*, and *TP53* in DLBCL cell lines.**

| Cell line | *BCL2*# | *BAX* | *TP53* |
|---|---|---|---|
| SUDHL4 Parental | P59T, S117R | No mutation | R273C |
| SUDHL4 Resistant | F104L/V*, P59T, S117R | No mutation | R273C |
| SUDHL6 Parental | T7S, I48F#, R129C | No mutation | Y234C |
| SUDHL6 Resistant | T7S, I48F#, R129C | No mutation | Y234C |
| SUDHL16 Parental | No mutation | No mutation | Heterozygous mutation: 39 bp insertion and 107 bp deletion |
| SUDHL16 Resistant | No mutation | No mutation | Heterozygous mutation: 39 bp insertion and 107 bp deletion |

*Heterozygous mutation in the BH3 binding domain (disrupts venetoclax binding)
#Mutation prevents BCL2 antibody binding to SUDHL4 (hamster mAb clone 6C8) or SUDHL6 (mouse mAb clone 124)

**Supplementary Table S4. Synergy analyses for combination treatments in DLBCL lines.**

| | Cell Line | Drug 1 | Drug 2 | ZIP | | Bliss | | H.S.A. | | Loewe | |
|---|---|---|---|---|---|---|---|---|---|---|---|
| | | | | Score | P-value | Score | P-value | Score | P-value | Score | P-value |
| Figure 2B, S1C | SUDHL4 Par | BCL2i | BCLXi | 18.0 | $6.3 \times 10^{-4}$ | 17.8 | $5.8 \times 10^{-4}$ | 24.7 | $1.2 \times 10^{-4}$ | 16.4 | $1.4 \times 10^{-3}$ |
| | SUDHL4 Par | BCL2i | MCL1i | 48.8 | $2.4 \times 10^{-7}$ | 49.1 | $2.4 \times 10^{-7}$ | 52.9 | $2.5 \times 10^{-7}$ | 51.8 | $1.2 \times 10^{-7}$ |
| | SUDHL4 Res | BCL2i | BCLXi | 8.4 | $8.8 \times 10^{-4}$ | 7.9 | $4.5 \times 10^{-3}$ | 9.6 | $5.2 \times 10^{-3}$ | 4.1 | $3.9 \times 10^{-2}$ |
| | SUDHL4 Res | BCL2i | MCL1i | 31.2 | $1.7 \times 10^{-5}$ | 31.2 | $1.8 \times 10^{-5}$ | 36.0 | $5.9 \times 10^{-6}$ | 26.0 | $2.5 \times 10^{-4}$ |
| | SUDHL6 Par | BCL2i | BCLXi | 37.1 | $1.0 \times 10^{-6}$ | 37.0 | $2.2 \times 10^{-6}$ | 48.6 | $9.4 \times 10^{-7}$ | 43.7 | $7.0 \times 10^{-9}$ |
| | SUDHL6 Par | BCL2i | MCL1i | 53.0 | $1.3 \times 10^{-5}$ | 55.0 | $2.5 \times 10^{-8}$ | 65.2 | $1.6 \times 10^{-21}$ | 42.9 | $1.6 \times 10^{-19}$ |
| | SUDHL6 Res | BCL2i | BCLXi | 14.8 | $3.5 \times 10^{-1}$ | 12.6 | $5.3 \times 10^{-1}$ | 13.7 | $5.4 \times 10^{-1}$ | 6.5 | $7.5 \times 10^{-1}$ |
| | SUDHL6 Res | BCL2i | MCL1i | 6.5 | $6.6 \times 10^{-1}$ | 3.0 | $8.7 \times 10^{-1}$ | 5.8 | $7.6 \times 10^{-1}$ | -7.6 | $7.3 \times 10^{-1}$ |
| | SUDHL16 Par | BCL2i | BCLXi | 14.0 | $4.8 \times 10^{-1}$ | 13.9 | $4.9 \times 10^{-1}$ | 19.5 | $3.7 \times 10^{-1}$ | 12.0 | $5.4 \times 10^{-1}$ |
| | SUDHL16 Par | BCL2i | MCL1i | 41.9 | $6.7 \times 10^{-4}$ | 42.1 | $1.5 \times 10^{-3}$ | 44.0 | $2.8 \times 10^{-3}$ | 37.6 | $7.5 \times 10^{-3}$ |
| | SUDHL16 Res | BCL2i | BCLXi | 0.4 | $9.6 \times 10^{-1}$ | -3.1 | $7.5 \times 10^{-1}$ | 3.1 | $7.1 \times 10^{-1}$ | 3.0 | $6.5 \times 10^{-1}$ |
| | SUDHL16 Res | BCL2i | MCL1i | 30.9 | $1.9 \times 10^{-1}$ | 30.3 | $2.2 \times 10^{-1}$ | 32.8 | $2.0 \times 10^{-1}$ | 24.6 | $2.6 \times 10^{-1}$ |
| Figure 2B, S1D | OCI-Ly10 | BCL2i | BCLXi | 25.5 | $4.0 \times 10^{-3}$ | 24.3 | $6.7 \times 10^{-3}$ | 27.2 | $2.4 \times 10^{-3}$ | 27.3 | $2.0 \times 10^{-3}$ |
| | OCI-Ly10 | BCL2i | MCL1i | 28.4 | $3.0 \times 10^{-5}$ | 27.6 | $3.7 \times 10^{-5}$ | 31.2 | $1.3 \times 10^{-5}$ | 31.4 | $1.2 \times 10^{-5}$ |
| | Toledo | BCL2i | BCLXi | 31.2 | $4.4 \times 10^{-3}$ | 31.0 | $1.1 \times 10^{-2}$ | 36.4 | $2.2 \times 10^{-2}$ | 32.8 | $3.5 \times 10^{-2}$ |
| | Toledo | BCL2i | MCL1i | 28.3 | $2.2 \times 10^{-3}$ | 28.0 | $5.1 \times 10^{-3}$ | 34.6 | $6.3 \times 10^{-3}$ | 27.4 | $4.8 \times 10^{-3}$ |
| Figure 2B, S1E | SUDHL2 | BCL2i | BCLXi | 11.1 | $1.3 \times 10^{-4}$ | 11.4 | $8.2 \times 10^{-5}$ | 19.5 | $2.7 \times 10^{-4}$ | 17.8 | $7.4 \times 10^{-4}$ |
| | SUDHL2 | BCL2i | MCL1i | 7.2 | $8.4 \times 10^{-3}$ | 7.5 | $5.2 \times 10^{-3}$ | 13.1 | $1.9 \times 10^{-5}$ | 5.4 | $5.5 \times 10^{-3}$ |
| | SUDHL5 | BCL2i | BCLXi | 11.8 | $9.4 \times 10^{-4}$ | 11.5 | $2.1 \times 10^{-3}$ | 14.6 | $3.5 \times 10^{-3}$ | 6.6 | $1.4 \times 10^{-1}$ |
| | SUDHL5 | BCL2i | MCL1i | 23.4 | $1.9 \times 10^{-3}$ | 22.8 | $3.4 \times 10^{-3}$ | 28.4 | $1.5 \times 10^{-3}$ | 15.5 | $2.3 \times 10^{-2}$ |
| | SUDHL8 | BCL2i | BCLXi | 10.4 | $8.7 \times 10^{-4}$ | 10.8 | $3.0 \times 10^{-4}$ | 13.5 | $3.7 \times 10^{-5}$ | 3.9 | $1.9 \times 10^{-1}$ |
| | SUDHL8 | BCL2i | MCL1i | 53.1 | $2.7 \times 10^{-6}$ | 52.9 | $8.4 \times 10^{-6}$ | 56.3 | $2.4 \times 10^{-6}$ | 52.8 | $1.7 \times 10^{-6}$ |
| | SUDHL10 | BCL2i | BCLXi | 16.0 | $8.2 \times 10^{-4}$ | 15.1 | $2.8 \times 10^{-3}$ | 16.1 | $3.3 \times 10^{-3}$ | 0.3 | $9.6 \times 10^{-1}$ |
| | SUDHL10 | BCL2i | MCL1i | 1.9 | $5.6 \times 10^{-1}$ | 1.9 | $5.9 \times 10^{-1}$ | 9.2 | $4.0 \times 10^{-4}$ | -0.5 | $8.1 \times 10^{-1}$ |
| | OCI-Ly3 | BCL2i | BCLXi | 24.1 | $4.5 \times 10^{-7}$ | 23.6 | $3.7 \times 10^{-7}$ | 25.7 | $1.7 \times 10^{-6}$ | 24.6 | $7.9 \times 10^{-7}$ |
| | OCI-Ly3 | BCL2i | MCL1i | 27.0 | $1.1 \times 10^{-8}$ | 26.8 | $7.0 \times 10^{-9}$ | 36.7 | $1.0 \times 10^{-8}$ | 36.0 | $1.6 \times 10^{-8}$ |
| Fig 4B | SUDHL6 Res | BCL2i | IDH2i | 20.0 | $6.0 \times 10^{-3}$ | 18.3 | $1.7 \times 10^{-2}$ | 18.7 | $1.9 \times 10^{-2}$ | 11.1 | $8.0 \times 10^{-2}$ |
| | SUDHL16 Res | BCL2i | IDH2i | 26.2 | $3.0 \times 10^{-3}$ | 26.2 | $2.9 \times 10^{-3}$ | 27.9 | $2.7 \times 10^{-3}$ | 9.5 | $1.3 \times 10^{-1}$ |
| Fig 4C | SUDHL6 Res | BCL2i | IDH2i | 28.1 | $8.0 \times 10^{-5}$ | 27.5 | $1.4 \times 10^{-4}$ | 31.6 | $1.0 \times 10^{-4}$ | 8.1 | $4.7 \times 10^{-3}$ |
| | SUDHL16 Res | BCL2i | IDH2i | 28.9 | $5.6 \times 10^{-5}$ | 28.6 | $7.0 \times 10^{-5}$ | 32.4 | $1.0 \times 10^{-4}$ | 24.1 | $1.8 \times 10^{-3}$ |

**Supplementary Table S5. Upregulated Hallmark pathways with FDR<0.05.**

| SUDHL6 Parental vs Resistant | | | |
|---|---|---|---|
| **PATHWAY NAME** | **NES** | **NOM p-value** | **FDR q-value** |
| HALLMARK_INTERFERON_ALPHA_RESPONSE | 3.3066053 | 0 | 0 |
| **HALLMARK_OXIDATIVE_PHOSPHORYLATION** | 3.109107 | 0 | 0 |
| HALLMARK_INTERFERON_GAMMA_RESPONSE | 2.9325926 | 0 | 0 |
| HALLMARK_COAGULATION | 2.236325 | 0 | 0.00597988 |
| HALLMARK_APICAL_JUNCTION | 2.2003255 | 0.001838235 | 0.006981376 |
| HALLMARK_MITOTIC_SPINDLE | 2.0006518 | 0.004081633 | 0.016969811 |
| HALLMARK_INFLAMMATORY_RESPONSE | 1.893132 | 0.006012024 | 0.026134815 |
| | | | |
| | | | |
| **SUDHL16 Parental vs Resistant** | | | |
| **PATHWAY NAME** | **NES** | **NOM p-value** | **FDR q-value** |
| HALLMARK_INTERFERON_GAMMA_RESPONSE | 5.812295 | 0 | 0 |
| HALLMARK_INTERFERON_ALPHA_RESPONSE | 4.5776544 | 0 | 0 |
| HALLMARK_TNFA_SIGNALING_VIA_NFKB | 3.4320908 | 0 | 0 |
| HALLMARK_ALLOGRAFT_REJECTION | 3.4005704 | 0 | 0 |
| HALLMARK_ADIPOGENESIS | 3.1016262 | 0 | 0 |
| **HALLMARK_OXIDATIVE_PHOSPHORYLATION** | 3.0908427 | 0 | 0 |
| HALLMARK_INFLAMMATORY_RESPONSE | 3.0253499 | 0 | 0 |
| HALLMARK_DNA_REPAIR | 2.9278455 | 0 | 0 |
| HALLMARK_HYPOXIA | 2.7757347 | 0 | 2.13E-04 |
| HALLMARK_P53_PATHWAY | 2.613183 | 0 | 1.92E-04 |
| HALLMARK_COAGULATION | 2.5499294 | 0 | 1.74E-04 |
| HALLMARK_IL2_STAT5_SIGNALING | 2.5256906 | 0 | 3.07E-04 |
| HALLMARK_E2F_TARGETS | 2.4007585 | 0 | 9.91E-04 |
| HALLMARK_IL6_JAK_STAT3_SIGNALING | 2.3666384 | 0 | 0.001037984 |
| HALLMARK_PROTEIN_SECRETION | 2.3120022 | 0 | 0.001204682 |
| HALLMARK_HEME_METABOLISM | 2.215449 | 0.003968254 | 0.00266815 |
| HALLMARK_PI3K_AKT_MTOR_SIGNALING | 2.2093172 | 0.001879699 | 0.0025112 |
| HALLMARK_UNFOLDED_PROTEIN_RESPONSE | 2.2057855 | 0 | 0.002706716 |
| HALLMARK_G2M_CHECKPOINT | 2.1873155 | 0 | 0.003340711 |
| HALLMARK_MITOTIC_SPINDLE | 2.1143682 | 0.006072875 | 0.005869198 |
| HALLMARK_ESTROGEN_RESPONSE_LATE | 2.0847096 | 0.004175365 | 0.006695852 |
| HALLMARK_KRAS_SIGNALING_DN | 1.9519016 | 0.009487666 | 0.015715774 |
| HALLMARK_MTORC1_SIGNALING | 1.9448671 | 0.006160164 | 0.01576256 |
| HALLMARK_COMPLEMENT | 1.8807902 | 0.023060797 | 0.020825384 |
| HALLMARK_MYOGENESIS | 1.8365586 | 0.017208412 | 0.024390116 |
| HALLMARK_EPITHELIAL_MESENCHYMAL_TRANSITION | 1.81746 | 0.015533981 | 0.025536034 |
| HALLMARK_NOTCH_SIGNALING | 1.7690649 | 0.009633912 | 0.03290506 |
| HALLMARK_APICAL_SURFACE | 1.722918 | 0.024242423 | 0.04083286 |
| HALLMARK_APOPTOSIS | 1.7107453 | 0.026584867 | 0.0419554 |

Red font indicates overlap between the two cell lines.

Adams et al Supplemental Figure S1 – part 1

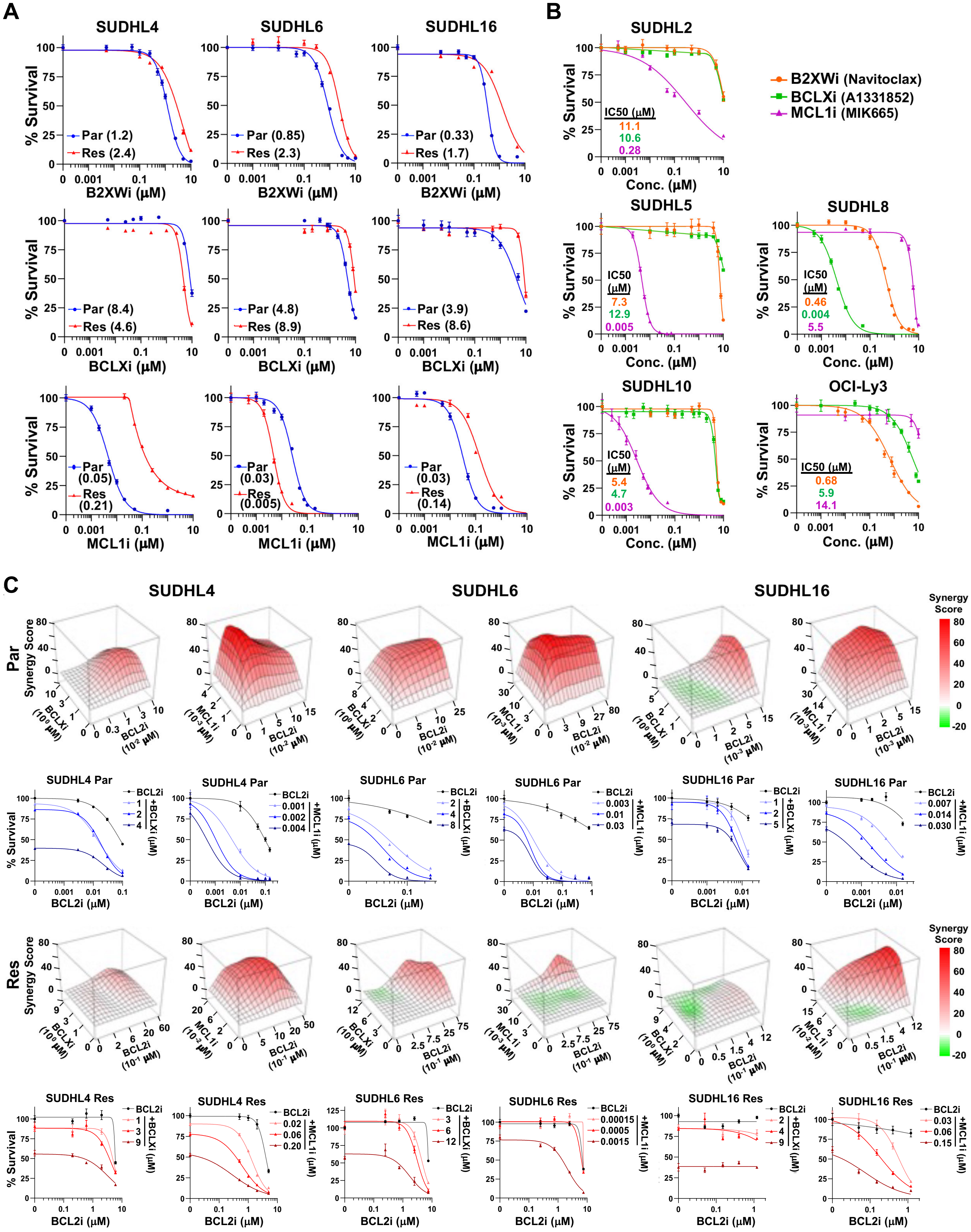

**Adams et al Supplemental Figure S1 – part 2**

**D**

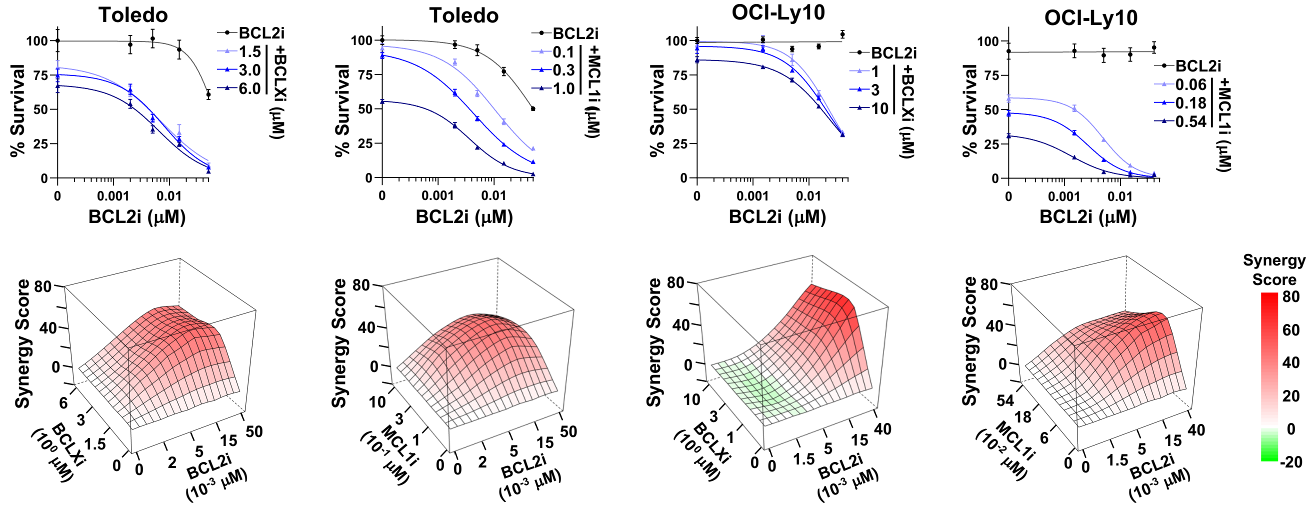


**E**

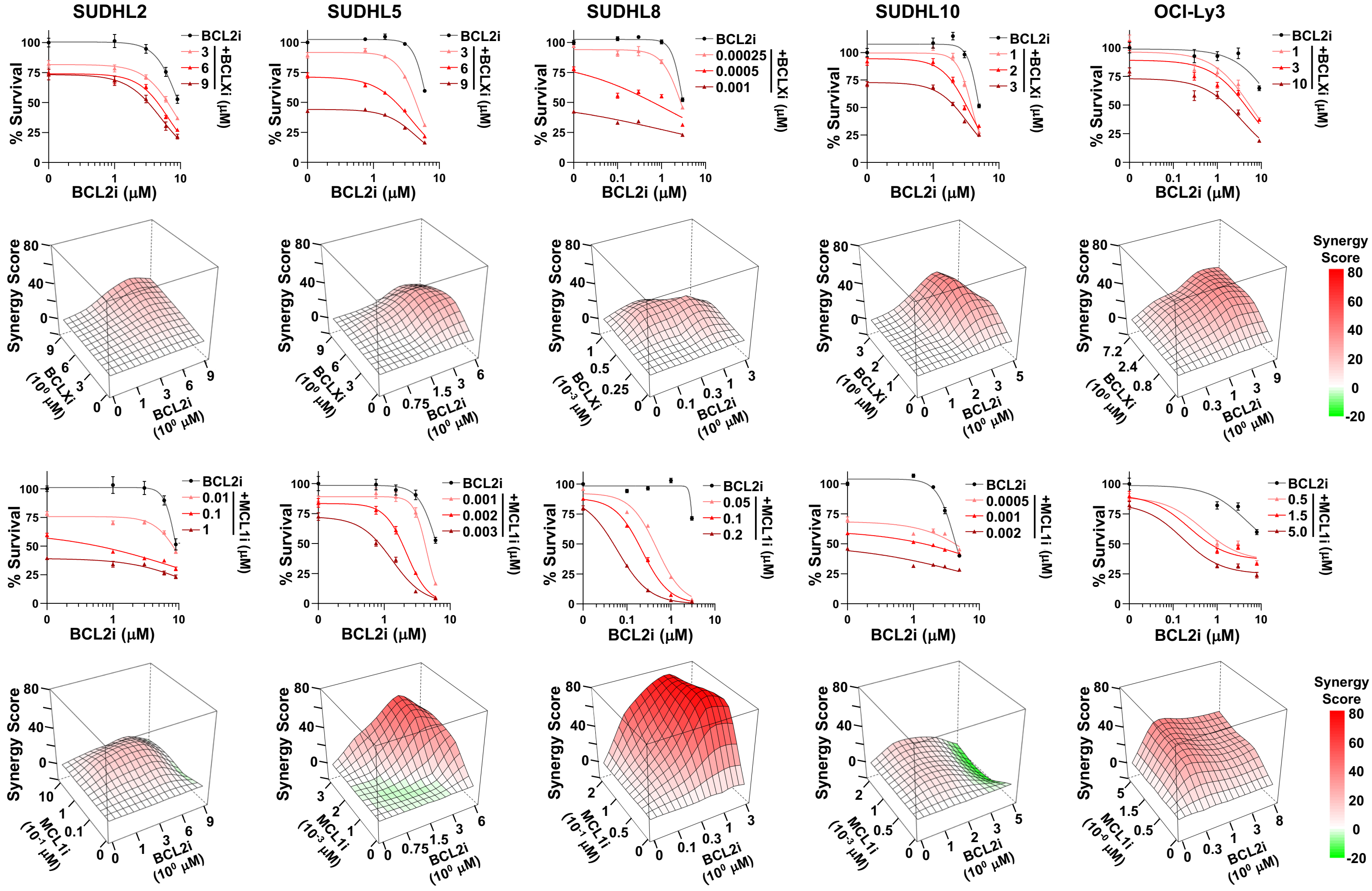

**Supplementary Figure S1. Synergy in DLBCL lines with combination treatment of venetoclax and BCLXi or MCLi.** A, B) Dose response curves (MTS, 48hrs, relative to DMSO vehicle control, quadruplicates, mean ±SEM) of navitoclax (B2XWi), A-1331852 (BCLXi), and MIK665 (MCL1i) in acquired venetoclax-resistant (Res) and parental (Par) DLBCL lines (A) and intrinsically venetoclax-resistant DLBCL lines (B). IC50s (μM) in parentheses (A) or indicated (B). C) ZIP synergy 3D plots (top) and MTS assays (bottom, 48hrs, relative to DMSO vehicle control, quadruplicates, mean ± SEM) for synergy evaluation of venetoclax (BCL2i) + BCLXi or MCL1i for the three parental and acquired venetoclax-resistant DLBCL lines. D, E) MTS assays (48hrs, relative to DMSO vehicle control, quadruplicates, mean ± SEM) for synergy evaluation of DLBCL lines treated with venetoclax (BCL2i) + BCLXi or MCL1i of D) two additional intrinsically venetoclax-sensitive DLBCL lines (top) and their ZIP 3D synergy plots (bottom) and E) intrinsically venetoclax-resistant DLBCL lines (top) and their ZIP 3D synergy plots (bottom). See Supplementary Table S4 for synergy scores from other synergy methods.

Adams et al Supplemental Figure S2

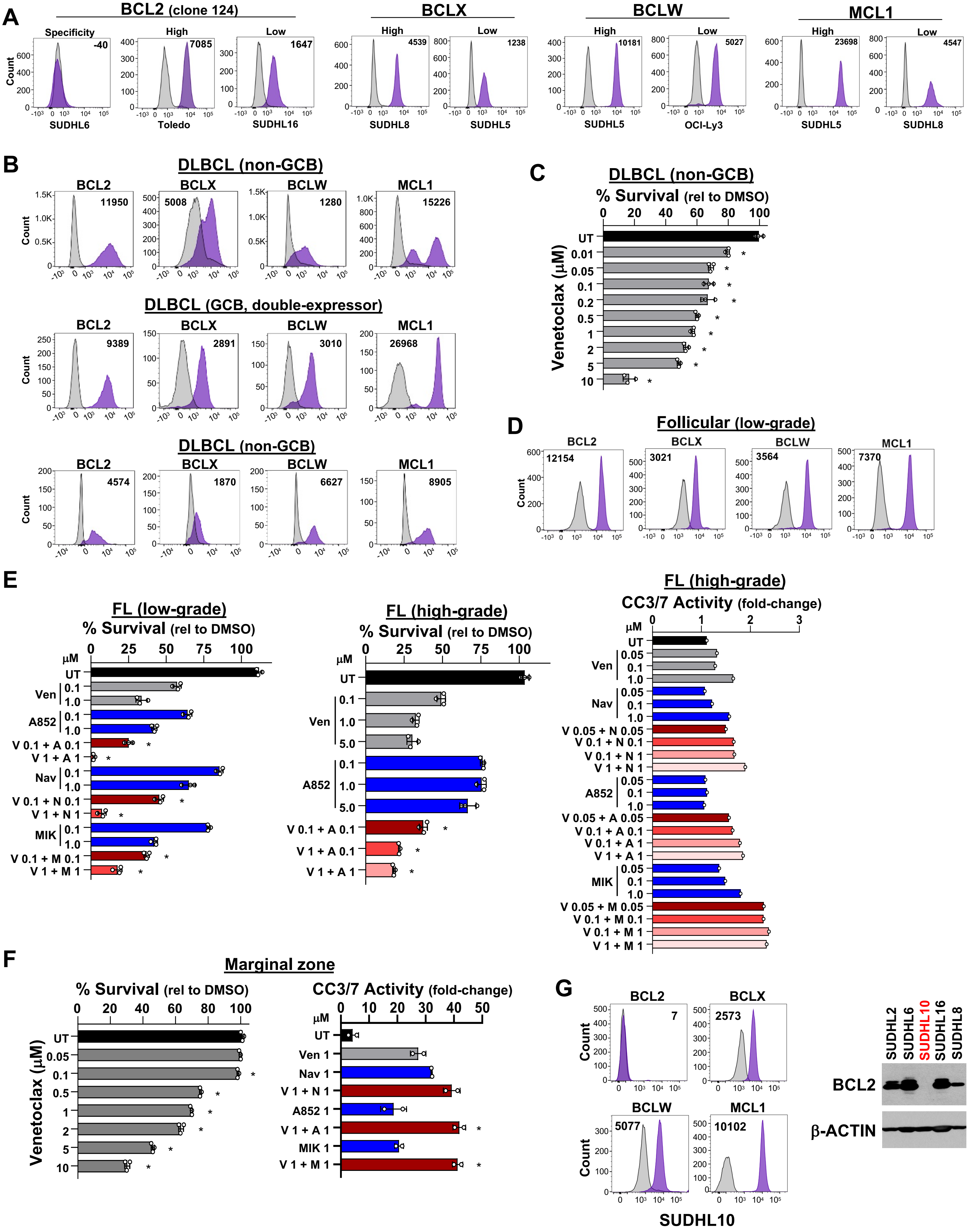

**Supplementary Figure S2. Intracellular levels of BCL2 family members and treatment of B-cell lymphoma patient samples with BCL2 family member inhibitors.** A, B) Intracellular flow cytometry of four anti-apoptotic BCL2 family members in DLBCL cell lines (A) and fresh patient samples of DLBCL (B). Representative histograms shown with median fluorescence intensity (MFI) after subtracting the isotype control MFI value. C) B cells were isolated from a fresh patient sample of DLBCL. Cell survival (MTS assay, triplicates, mean ± SD) were measured 12hrs after treatment with venetoclax or DMSO vehicle control or left untreated (UT); *$P<0.0001$. D) Intracellular flow cytometry as described in A above in a fresh patient sample of follicular lymphoma. E, F) B cells were isolated from fresh patient samples follicular lymphoma (E) and marginal zone lymphoma (F). Cell survival (MTS assay, triplicates, mean ± SD) and/or Caspase-3/7 activity (mean ± SD) were measured 6 or 12hrs after treatment with the compounds indicated. Venetoclax (Ven, V), navitoclax (Nav, N), A-1331852 (A852, A), MIK665 (MIK, M), and untreated (UT). For E *$P<0.001$ (middle) and *$P<0.01$ (left), and F *$P<0.035$ (left) and *$P<0.01$ (right), comparing each concentration used in the combination treatment to the same concentration of each single agent. G) Using two different antibodies against two different epitopes of BCL2, intracellular detection of BCL2 (left) and Western blotting (right, overexposed purposefully) was performed on SUDHL10 cells. Other BCL2 family members (left) and other DLBCL cell lines (right) were also evaluated at the same time.

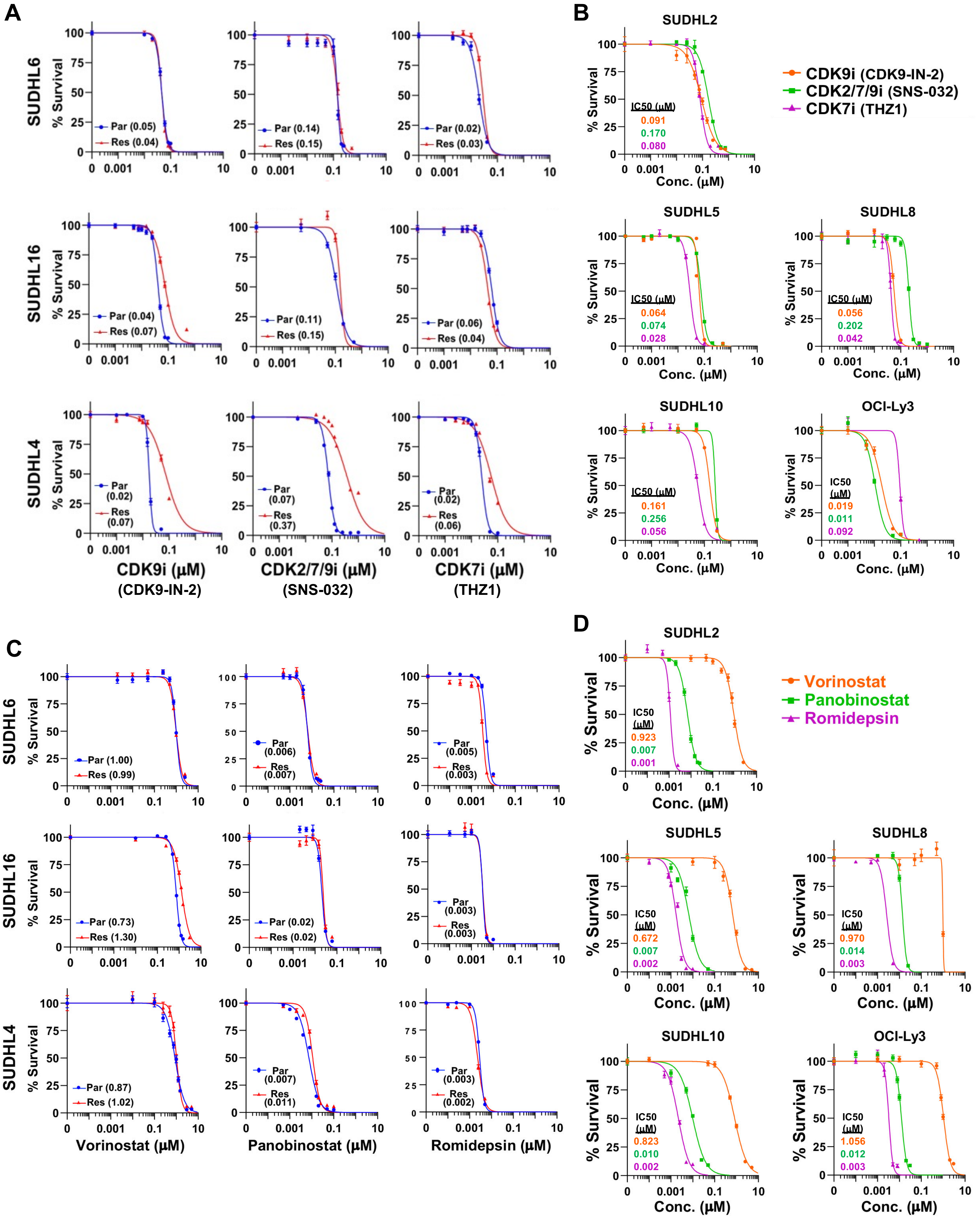
A
SUDHL6
% Survival
Par (0.05)
Res (0.04)
Par (0.14)
Res (0.15)
Par (0.02)
Res (0.03)
SUDHL16
Par (0.04)
Res (0.07)
Par (0.11)
Res (0.15)
Par (0.06)
Res (0.04)
SUDHL4
Par (0.02)
Res (0.07)
Par (0.07)
Res (0.37)
Par (0.02)
Res (0.06)
CDK9i (µM)
(CDK9-IN-2)
CDK2/7/9i (µM)
(SNS-032)
CDK7i (µM)
(THZ1)
B
SUDHL2
CDK9i (CDK9-IN-2)
CDK2/7/9i (SNS-032)
CDK7i (THZ1)
IC50 (µM)
0.091
0.170
0.080
Conc. (µM)
SUDHL5
IC50 (µM)
0.064
0.074
0.028
SUDHL8
IC50 (µM)
0.056
0.202
0.042
SUDHL10
IC50 (µM)
0.161
0.256
0.056
OCI-Ly3
IC50 (µM)
0.019
0.011
0.092
C
SUDHL6
Par (1.00)
Res (0.99)
Par (0.006)
Res (0.007)
Par (0.005)
Res (0.003)
SUDHL16
Par (0.73)
Res (1.30)
Par (0.02)
Res (0.02)
Par (0.003)
Res (0.003)
SUDHL4
Par (0.87)
Res (1.02)
Par (0.007)
Res (0.011)
Par (0.003)
Res (0.002)
Vorinostat (µM)
Panobinostat (µM)
Romidepsin (µM)
D
SUDHL2
Vorinostat
Panobinostat
Romidepsin
IC50 (µM)
0.923
0.007
0.001
SUDHL5
IC50 (µM)
0.672
0.007
0.002
SUDHL8
IC50 (µM)
0.970
0.014
0.003
SUDHL10
IC50 (µM)
0.823
0.010
0.002
OCI-Ly3
IC50 (µM)
1.056
0.012
0.003

**Supplementary Figure S3. CDK7/9i and HDACi circumvent venetoclax-resistant DLBCL.** A-D) Acquired venetoclax-resistant (Res) and parental (Par) DLBCL lines (A and C) and intrinsically venetoclax-resistant DLBCL lines (B and D) treated with CDK7/9 (A and B) or HDAC (C and D) inhibitors indicated. MTS assays performed (48hrs, quadruplicates, relative to DMSO vehicle control, mean ±SEM). IC50s are either indicated (B and D) or in parentheses (A and C, μM).

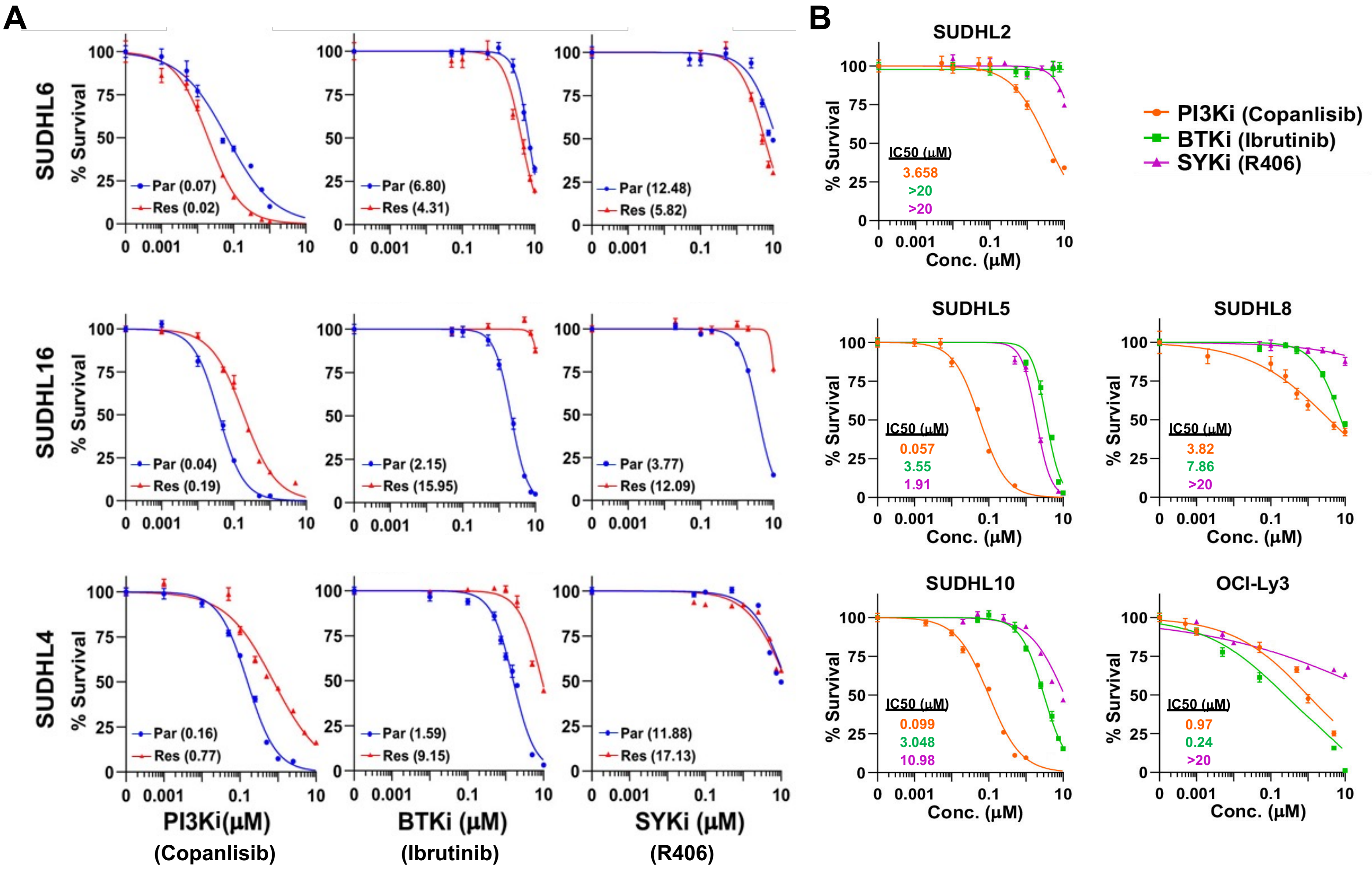
A
B
SUDHL6
SUDHL16
SUDHL4
% Survival
Par (0.07)
Res (0.02)
Par (6.80)
Res (4.31)
Par (12.48)
Res (5.82)
Par (0.04)
Res (0.19)
Par (2.15)
Res (15.95)
Par (3.77)
Res (12.09)
Par (0.16)
Res (0.77)
Par (1.59)
Res (9.15)
Par (11.88)
Res (17.13)
PI3Ki(µM)
(Copanlisib)
BTKi (µM)
(Ibrutinib)
SYKi (µM)
(R406)
SUDHL2
IC50 (µM)
3.658
>20
>20
SUDHL5
IC50 (µM)
0.057
3.55
1.91
SUDHL8
IC50 (µM)
3.82
7.86
>20
SUDHL10
IC50 (µM)
0.099
3.048
10.98
OCI-Ly3
IC50 (µM)
0.97
0.24
>20
Conc. (µM)
PI3Ki (Copanlisib)
BTKi (Ibrutinib)
SYKi (R406)

**Supplementary Figure S4. BCRi can decrease survival of venetoclax-resistant DLBCL.** BCRi [copanlisib (PI3Ki), ibrutinib (BTKi), or R406 (SYKi) dose response curves (48hrs, MTS assay, relative to DMSO vehicle control, quadruplicates, mean ± SEM) for (A) parental (Par) and acquired venetoclax-resistant (Res) DLBCL cell lines or (B) intrinsically venetoclax-resistant DLBCL lines. IC50s are either in parentheses (A, μM) or indicated (B).

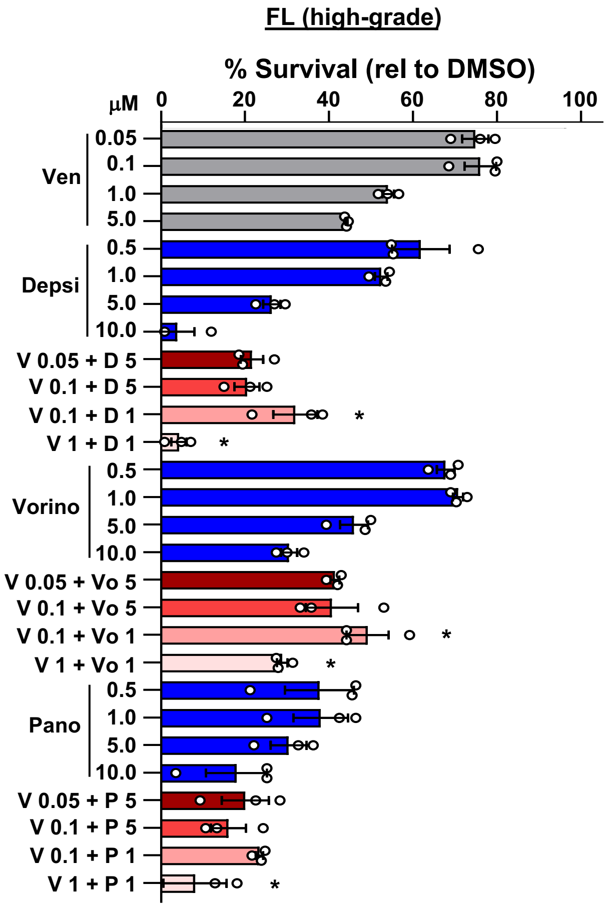
FL (high-grade)
% Survival (rel to DMSO)
μM
0
20
40
60
80
100
Ven
0.05
0.1
1.0
5.0
Depsi
0.5
1.0
5.0
10.0
V 0.05 + D 5
V 0.1 + D 5
V 0.1 + D 1
V 1 + D 1
Vorino
0.5
1.0
5.0
10.0
V 0.05 + Vo 5
V 0.1 + Vo 5
V 0.1 + Vo 1
V 1 + Vo 1
Pano
0.5
1.0
5.0
10.0
V 0.05 + P 5
V 0.1 + P 5
V 0.1 + P 1
V 1 + P 1
*
*
*
*
*

**Supplementary Figure S5. HDACi synergizes with venetoclax in follicular lymphoma.** MTS assay of enriched B-cells from a fresh follicular lymphoma patient sample following treatment with venetoclax (Ven, V), romidepsin (Depsi, D), vorinostat (Vorino, Vo), and/or panobinostat (Pano, P); 24hrs, triplicates, relative to DMSO vehicle control, mean ± SD, *P<0.05 (comparing each combination treatment to both single agent treatments at the same concentrations).
